# BrainScape: An open-source framework for integrating and preprocessing anatomical MRI datasets

**DOI:** 10.1162/IMAG.a.944

**Published:** 2025-10-22

**Authors:** Muhammad Nabi Yasinzai, Remika Mito, Mangor Pedersen

**Affiliations:** Department of Psychology & Neuroscience, Auckland University of Technology, Auckland, New Zealand; Department of Psychiatry, University of Melbourne, Melbourne, Victoria, Australia

**Keywords:** BrainScape, deep learning MRI, multimodal MRI dataset, MRI preprocessing, MRI data integration, and MRI data pooling

## Abstract

MRI has revolutionized our ability to investigate and understand brain structure and function in health and disease. A large amount of MRI data is widely available to researchers, both from large-scale multi-site consortia and smaller site-specific datasets. This wealth of MRI data offers opportunities to advance our understanding of the brain, particularly through machine learning and deep learning approaches that rely on large sample sizes to reveal complex associations between brain organization and its behavioral and clinical associations. Many large-scale initiatives provide extensive datasets with sufficient statistical power to support reproducibility, but reproducibility alone does not ensure clinical relevance or broad generalizability due to narrow demographic representations and minimized dataset variability. Recent work highlights the need to embrace dataset variability and open-science collaborations for pooling heterogeneous datasets. Nevertheless, effectively integrating these diverse resources remains a significant challenge. Inconsistencies in organization, data formatting, acquisition protocols, and metadata remain, especially for smaller, site-specific datasets, despite ongoing efforts within the neuroimaging community to standardize data sharing practices. To address these issues, we introduce BrainScape: a curated collection of 160 publicly available MRI datasets packaged with an open-source, plugin-based Python framework that automates the download, organization, preprocessing, and demographic attachment of the MRI data. Each individual dataset includes a detailed configuration file capturing all dataset-specific parameters, enabling other researchers to regenerate the BrainScape dataset. The current BrainScape dataset integrates 160 datasets, encompassing a total of 27227 subjects and 46583 multimodal MRI scans after quality control. The BrainScape framework’s pipeline effectively aggregates these heterogeneous datasets while preserving the original dataset structure and demographic details. Its modular design allows integration into data pipelines, supporting large-scale studies involving diverse cohorts and targeted research on rare phenotypes. BrainScape framework employs an easy-to-use plugin-based architecture with distinct modules for data downloading, file mapping, validation, preprocessing, and demographics attachment. Furthermore, each MR image can be traced to its source project and repository, and subjects excluded from datasets are documented in dedicated dataset-specific configuration files, providing transparent and reproducible exclusion criteria. BrainScape dataset includes multiple MRI modalities such as T1-weighted (T1w), T2-weighted (T2w), gadolinium-enhanced T1-weighted (T1Gd), and fluid-attenuated inversion recovery (FLAIR) from diverse sources and integrates key demographic fields, such as age, sex, and handedness, for large-scale studies. This unified workflow reduces manual labor and minimizes the risk of data duplication and biases. By providing automated, transparent, and configurable workflows, BrainScape hopes to address open science challenges, accelerate data-driven investigations, and promote inclusivity and reproducibility in neuroscience research.

## 1 Introduction

Magnetic resonance imaging (MRI) has revolutionized our understanding of brain structure and function, enabling non-invasive studies of brain development, plasticity, and pathology in healthy and clinical cohorts (Katti et al., 2011; Mills & Tamnes, 2014). MRI research has advanced through the adoption of open science practices, especially with the open sharing of MRI data (Di Martino et al., 2014; Miller et al., 2016; Snoek et al., 2021; Van Essen et al., 2013). Large-scale initiatives are valuable resources for population-level analyses and have contributed to big data efforts in neuroimaging, with some examples including the Human Connectome Project (HCP; Van Essen et al., 2013), CORR (Gorgolewski et al., 2017), FC1000 (Biswal et al., 2010), ABCD (Casey et al., 2018), GSP (Holmes et al., 2015), ADNI (Mueller et al., 2005), and UK Biobank (Miller et al., 2016). Thanks to the open science ethos within the neuroimaging community, thousands of MRI datasets are now publicly available, facilitating research across various aspects of health and disease. Large-scale consortia are valued for their consistent acquisition protocols, enriched demographic details, and broad population coverage, while smaller specialized datasets remain critical for studying neurological and psychiatric disorders (Fajardo-Valdez et al., 2024; Gibson et al., 2024; Soler-Vidal et al., 2022).

Large neuroimaging datasets require substantial time and resources. For example, Horien et al. (2021) found that two or three researchers typically require about 6 to 9 months to download and process raw data from thousands of participants. Without an automated framework, researchers often invest considerable effort in downloading, ad hoc organizing, preprocessing, and performing quality control. This manual process can also tie investigators to previously preprocessed pipelines, restricting the scope of subsequent studies, as reprocessing large datasets again requires enormous effort (Horien et al., 2021). Additionally, careful organization and comprehensive documentation of every processing step are essential for reproducibility. By adopting widely accepted standards such as the Brain Imaging Data Structure (BIDS) and maintaining detailed records of software versions, code, and workflow decisions, researchers can ensure that other investigators can precisely replicate their study pipeline (Horien et al., 2021; White et al., 2022).

Large-scale consortia provide extensive sample sizes, offering the statistical power necessary to ensure the replicability of research findings, thereby effectively reducing false positives and minimizing inflated effect sizes in studies of brain–behavior associations and phenotype predictions (Marek & Laumann, 2024). While reproducibility establishes an essential baseline for validating findings, it alone does not ensure clinical utility or true generalizability (Kiar et al., 2024). Furthermore, these large datasets may lack generalizability due to demographic biases and over-representation of specific populations, limiting their applicability in broader clinical and real-world contexts (Marek & Laumann, 2024; Yang et al., 2024). In addition, many large consortium neuroimaging initiatives intentionally minimize dataset variability, creating harmonization that does not exist in real-world scenarios (Adkinson et al., 2024). Moreover, some large-scale MRI data-sharing initiatives such as UK Biobank, ABCD, ADNI, and GSP may require access fees or administrative approvals (Casey et al., 2018; Holmes et al., 2015; Miller et al., 2016; Mueller et al., 2005). These policies are intended to ensure responsible data use, protect participant confidentiality, and uphold ethical standards (White et al., 2022). However, while they help safeguard personal information, they may also create accessibility barriers for researchers with limited administrative support or funding. Another concern is data decay, which occurs when the repeated analysis of the same high-profile datasets leads to overfitting and reduces the generalizability of findings over time (Horien et al., 2021; W. H. Thompson et al., 2020).

Meanwhile, thousands of smaller, specialized, and openly accessible datasets provide an alternative source of MRI data for neuroscientists who may not otherwise have the resources to acquire data or access data from large-scale repositories. These MRI datasets are typically defaced and exclude sensitive demographic or genetic details, thereby minimizing privacy concerns. While they generally lack the richly characterized cohorts found in many of the large-scale repositories, such smaller datasets are still valuable in augmenting larger datasets for data-driven analyses. These smaller, specialized “boutique” datasets usually focus on specific brain disorders and targeted research studies. A promising way forward lies in open-science collaborations that combine diverse, heterogeneous datasets, enhancing both sample diversity and statistical power, and thereby improving reproducibility and generalizability of predictive brain–behavior associations (Adkinson et al., 2024; Marek & Laumann, 2024; Yang et al., 2024). Pooling these specialized datasets with large-scale consortia can broaden clinical relevance, mitigate data decay, prevent overfitting, and improve generalizability, thus enabling more advanced, data-driven analyses for diverse neurological conditions (Adkinson et al., 2024; Horien et al., 2021; Marek & Laumann, 2024; Yang et al., 2024). Both Adkinson et al. (2024) and Kiar et al. (2024) emphasize the importance of embracing dataset variability, including differences in demographics, scanner hardware, clinical status, and acquisition protocols, to build more robust and transferable models.

However, these resources are dispersed across multiple repositories and stored in incompatible formats, leading to fragmentation that complicates data pooling, for curating enriched multicentric datasets (Dishner et al., 2024). Furthermore, variability across multiple sites and scanners introduces biases that can significantly impact downstream analyses. Reducing multi-site scanner variability in MRI datasets is an active area of research, with harmonization techniques such as ComBat increasingly employed to mitigate these differences and enhance the reliability of multi-site studies (Fortin et al., 2017, 2018). Although harmonization techniques are increasingly employed to remove these differences, they can also remove real-world variability, which is often present in clinical settings. Adkinson et al. (2024), for example, demonstrated that unharmonized developmental samples can produce robust cross-dataset predictions, occasionally outperforming models trained within a single harmonized dataset. Compounding these challenges is the lack of standardized metadata on demographics and clinical variables, which complicates the merging of smaller specialized datasets in a reproducible manner (Pomponio et al., 2019).

However, the demand for machine learning and artificial intelligence applications in neuroimaging has rapidly increased. Advanced deep learning models require large, diverse, and well-annotated datasets to effectively train models capable of generalizing across populations, scanners, and clinical conditions (Dishner et al., 2024). Models trained on narrowly selected samples risk shortcut learning, where the model captures associations between brain and unintended confounding variables rather than underlying brain–behavior relationships (Marek & Laumann, 2024; Yang et al., 2024). Additionally, distribution shifts across populations and imaging protocols can undermine an AI model’s fairness and generalizability, particularly when models rely on demographic shortcuts, leading to biased and unreliable predictions (Yang et al., 2024). Pooling data from multiple datasets is a promising strategy to meet these requirements, enabling researchers to leverage cutting-edge artificial intelligence and deep learning approaches with enhanced generalizability. However, assembling and integrating such datasets remain challenging due to data fragmentation, limited data access, inconsistent annotation protocols, heterogeneous acquisition parameters, and demographic variability (Goldfarb & Teodoridis, 2022; Pomponio et al., 2019).

Several open neuroimaging platforms, including OpenNeuro and the NeuroImaging Tools and Resources Collaboratory (NITRC), have emerged to facilitate data sharing and standardization (Buccigrossi et al., 2008; Markiewicz et al., 2021). OpenNeuro (https://openneuro.org/) has become one of the largest repositories, hosting more than 1200 public datasets with free access and most datasets under Creative Commons Zero (CC0) licensing. This makes it an ideal choice for pooling datasets to increase their size and heterogeneity. Furthermore, OpenNeuro utilizes the robust Brain Imaging Data Structure (BIDS) format, which standardizes data organization and supports reproducible workflows, thereby reducing manual labor in data handling (Markiewicz et al., 2021). Researchers aiming to collate these smaller, specialized repositories often must resort to ad hoc scripts to reconcile directory structures, manage irregular metadata, and ensure consistent preprocessing steps, such as skull stripping, normalization, or anatomical registration.

To overcome these challenges and enhance data-driven research, we introduce BrainScape, which consists of two linked components: (i) the *BrainScape framework*, an open-source, plugin-based Python workflow, and (ii) the *BrainScape dataset*, a curated collection of 160 publicly available MRI repositories distributed as dataset-specific configuration files. Each dataset is represented by a comprehensive configuration file that specifies precise instructions required by the framework for downloading, organizing, preprocessing, and demographic annotation. By integrating this flexible software framework with dataset-specific configurations, BrainScape enables researchers to reproduce analysis-ready datasets through a consistent and transparent workflow. Our approach directly addresses the objective of improving reproducibility and generalizability highlighted by Adkinson et al. (2024), Kiar et al. (2024), Marek and Laumann (2024), and Yang et al. (2024). BrainScape framework allows combining smaller, specialized datasets with large-scale collations, allowing generalization across diverse populations as well as clinical cohorts. BrainScape maintains the integrity of each source dataset’s structure and metadata, ensuring no original information is lost or any bias is introduced from duplicate images. It keeps every MR image linked to its original source, retains a record of excluded subjects, and enforces standardized methods to ensure reproducibility. BrainScape significantly reduces the typical workload associated with downloading, organizing, and preprocessing MRI data from thousands of participants, by automating the entire workflow for integrating and preprocessing datasets. The BrainScape framework offers researchers a rapid start while still allowing complete workflow control. Moreover, the software framework allows dynamic customization, enabling researchers to incorporate new datasets, demographic information, and specialized preprocessing modules without the overhead of reworking the codebase. Researchers can share their BrainScape workflow configurations and associated plugins, allowing others to replicate and recreate preprocessed images locally. This approach avoids redistributing derivative datasets, while ensuring reproducible pipelines across different research groups. The BrainScape framework is built upon a plugin-based architecture, designed to:

- **Automate data handling:** Automatically download raw MRI scans, regardless of format, and map them into a standardized structure via a mapping plugin. This hands-free workflow minimizes manual intervention and reduces human error.
- **Demographic metadata integration:** Employ a YAML-based schema to attach relevant demographic fields (e.g., age, sex, race, handedness, clinical status) to each subject, enabling comprehensive, population-specific analyses.
- **Reproducibility:** Enforce transparent and traceable data processing pipelines through configurable default and dataset-specific parameters, ensuring that all operations are traceable and the same operations are applied uniformly across multiple datasets.
- **Extensibility:** Maintain a modular design that allows the integration of new data sources, demographic fields, and specialized workflows for data handling and preprocessing without overhauling the existing codebase. Each dataset’s configuration is stored separately, allowing for tailored updates and corrections.

By consolidating multiple open datasets and enforcing standardized methods, BrainScape reduces fragmentation, supports large-scale MRI research, and automates repetitive data-handling tasks. This enables researchers to focus on higher-level analyses such as structural biomarker discovery, or machine learning applications. Furthermore, we have kept track of the licensing and usage permissions of every dataset to ensure that ethical standards are upheld and proprietary data are respected when aggregating resources from various repositories. To properly credit each source repository included in BrainScape’s dataset, we maintain a dedicated “README” file that identifies each dataset’s source, cites relevant publications, and provides direct links to original resources. In this paper, we describe the BrainScape framework’s architecture and functionalities, along with details on the BrainScape dataset, aiming to empower the neuroscience community to build inclusive, large-scale datasets that facilitate robust computational approaches and advance our understanding of brain structure and function.

## 2 Methodology

In this section, we describe the plugin-based architecture of BrainScape’s framework, designed to systematically collect, standardize, and preprocess anatomical MRI data. We discuss its modular structure by explaining the functions and interdependencies of each module.

### 2.1 Overall framework design

The BrainScape framework is developed in Python and employs a configurable plugin-based architecture to allow flexible substitution of plugins without updating the codebase. Each core operation, such as dataset downloading, file mapping, and preprocessing, is encapsulated in an independent plugin that can be configured or replaced at runtime. Meanwhile, the BrainScape dataset includes a curated set of dataset-specific configuration files for 160 diverse datasets, each specifying the combination of downloader, mapper, and preprocessing plugins required for a given dataset, along with all necessary parameter settings. This design allows datasets with diverse sources, file structures, and acquisition protocols to be seamlessly integrated into a unified workflow.

**Fig. 1.**
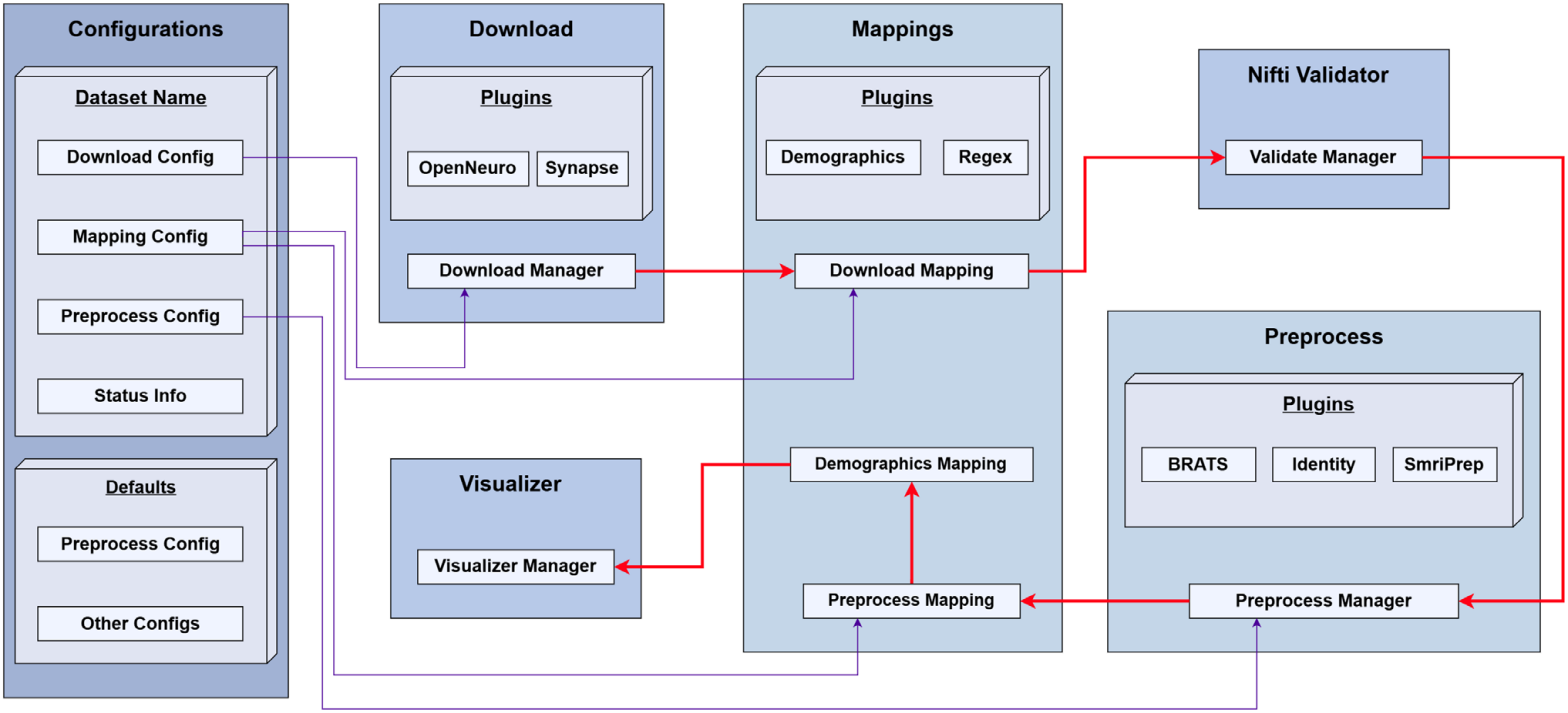
BrainScape Framework architecture.

Each plugin is responsible for a specific task, such as downloading MRI data from a given repository, performing MRI preprocessing, or mapping the downloaded files to a standardized JSON record (Fig. 1). Every pipeline stage (downloading, mapping, and preprocessing) has an associated abstract base class that exposes a minimal, stage-specific API (e.g., download(), map(), or preprocess()). Concrete implementations, such as “OpenNeuroDownloader” and “SynapseDownloader,” inherit from the “DownloaderPlugin” base class and override these methods. Plugins for each stage are organized into dedicated directories, containing the base class and its associated plugins; for instance, all downloader plugins reside in “download/downloader/” alongside the downloader base class.

At run time, BrainScape employs a “PluginLoader” for dynamic class loading. At the start of every workflow stage, the plugin loader scans the designated directories and registers each available plugin under its declared name (e.g., “OpenNeuroDownloader” or “SynapseDownloader” for downloader plugins). When the workflow enters a particular stage, it consults the dataset-specific JSON configuration, instantiates the plugin whose declared name matches the configuration entry, passes the relevant parameters, and invokes the plugin’s API method. Plugin selection is performed independently for each dataset and is driven entirely by its dataset-specific configuration, so introducing or switching plugins requires no changes to the core code.

This architecture makes the BrainScape framework intrinsically extensible. Adding a new plugin is as simple as creating a Python file that inherits the appropriate base class and placing it in the correct plugin directory. The new plugin will then be discovered automatically, and researchers can easily swap out or introduce new plugins (e.g., new preprocessing pipelines) customized to meet their specific research needs. Key objectives of the BrainScape framework include:

**Automation:** Minimizing manual labor by automating dataset downloads, file mapping, and organization, demographics attachment, and data preprocessing in a single pipeline.

**Standardization:** Maintaining a common mapping record and a standardized preprocessing pipeline across all datasets, while allowing users the flexibility to incorporate dataset-specific preprocessing or other customized plugins as needed.

**Extensibility:** Facilitating seamless integration of new data sources, additional preprocessing pipelines, and integration of new modules or plugins.

**Reproducibility:** Ensuring consistent execution and traceability by recording dataset-specific download, mapping, and processing parameters in JSON configuration files, thus facilitating accurate and reliable replication of the workflow.

### 2.2 Plugin-based architecture

Figure 1 illustrates the architecture of the BrainScape framework. At the core of this framework is a pipeline workflow that handles the sequential execution of the modules, such as downloading, mapping, and preprocessing (depicted by the red arrows in Fig. 1). This framework allows flexible selection and configuration of the plugins for each step, which can be tailored for each dataset via specific configuration files. The configurations integrate both default settings and dataset-specific overrides, maintaining consistency across datasets while allowing the flexibility to customize the pipeline for each dataset individually.

The pipeline workflow coordinates each module’s tasks, ensuring a structured flow of data and synchronized processing. **Downloader Plugins**, such as the OpenNeuro downloader and Synapse downloader, are used to download MRI data from target MRI databases and repositories. The OpenNeuro downloader uses the Amazon S3 CLI tool to download datasets from OpenNeuro (Markiewicz et al., 2021) and other large-scale databases such as the Human Connectome Project (HCP) (Van Essen et al., 2013). Similarly, the Synapse Downloader plugin retrieves MRI datasets from synapse.org. These plugins preserve each downloaded dataset’s original folder structure, simplifying comparisons after the datasets are obtained. **Mapper Plugins** (e.g., RegexMapper) then map the downloaded dataset files to a standardized JSON record, accommodating differences in dataset structure, organization, file formats, and MRI modalities based on user-defined, dataset-specific configuration settings. **Validator Plugins**, such as the NIfTI Validator, check each MRI file from all of the datasets to detect potential errors early, thereby helping the workflow maintain high data quality standards.

At the next stage of the pipeline, MRI preprocessing is carried out by configurable **Preprocessor Plugins**, each specialized for distinct imaging scenarios and requirements. Currently, the BrainScape framework supports three primary preprocessing plugins: BraTS, sMRIPrep, and Identity. The BraTS preprocessor plugin implements a preprocessing pipeline closely aligned with the well-known Brain Tumor Segmentation challenge (BraTS) dataset preprocessing protocol (Menze et al., 2014) by employing the BrainLes-Preprocessing Python package (v0.4.0), publicly available at https://github.com/BrainLesion/preprocessing. This pipeline consists of sequential preprocessing steps, including modality co-registration (aligning modalities such as T2-weighted, gadolinium-enhanced T1-weighted (T1Gd), and fluid-attenuated inversion recovery (FLAIR) images to a central T1-weighted modality), rigid-body registration to the SRI-24 (Rohlfing et al., 2010) atlas, skull stripping, and intensity normalization. The BrainLes-Preprocessing Python package utilizes the Advanced Normalization Tools (ANTs) for atlas registration and modality co-registration (Tustison et al., 2021). Given the heterogeneous nature of datasets aggregated in BrainScape, we implemented a configurable priority-based selection of the central modality, with the default priority order set to [T1w, T2w, T1Gd, FLAIR]. For brain extraction within the BraTS pipeline, BrainLes-Preprocessing employs “HD-BET” (Isensee et al., 2019), an artificial neural network-based tool known for robust, rapid brain extraction across diverse clinical MRI sequences (including T1w, T1Gd, T2w, and FLAIR modalities), pathologies, and scanner parameters. The HD-BET tool is utilized for brain extraction due to its superior accuracy and speed (typically less than 5 seconds per MRI sequence on a GPU) compared with widely used tools such as FSL’s BET and AFNI’s 3dSkullStrip (Isensee et al., 2019; Smith, 2000). The sMRIPrep plugin wraps the latest containerized version of the well-established sMRIPrep anatomical MRI preprocessing pipeline (Esteban et al., 2021), openly accessible at https://www.nipreps.org/smriprep/. Within sMRIPrep, anatomical images are aligned using ANTs’ antsRegistration algorithm, enabling spatial normalization to standard templates such as MNI152 (Avants et al., 2008, 2011). The BrainScape sMRIPrep plugin runs the latest stable sMRIPrep Docker image (nipreps/smriprep:latest) with its default ANTs-based skull-stripping workflow, while disabling FreeSurfer surface reconstruction, Multimodal Surface Matching (MSM), and submillimeter reconstructions (Esteban et al., 2021). Lastly, the Identity preprocessor plugin serves as a pass-through solution designed explicitly for datasets that have already undergone preprocessing or skull stripping. This approach ensures previously processed data remain unaltered, significantly simplifying the integration of diverse, pre-curated datasets into BrainScape. For each dataset within the BrainScape dataset collation, a dataset-specific configuration file selects the appropriate preprocessing plugin. It also supplies all necessary parameters for that plugin, enabling each dataset to be preprocessed independently.

All preprocessing plugins currently available handle MRI sessions independently; longitudinal pipelines were deliberately omitted because our primary goal is to prepare individual T1w, T2w, FLAIR, and T1Gd volumes for downstream deep learning models. The sMRIPrep plugin was included because sMRIPrep is one of the most widely used structural MRI preprocessing pipelines, while the BraTS and Identity plugins align directly with our subsequent studies on AI-based brain tumor segmentation. Nevertheless, BrainScape’s modular, plugin-based architecture supports the addition of longitudinal or other specialized preprocessing pipelines based on the research requirements. To facilitate and encourage community-driven extensibility, we provide comprehensive tutorials on the BrainScape GitHub repository (https://github.com/yasinzaii/BrainScape), illustrating how researchers can develop custom plugins to meet diverse research requirements.

After preprocessing, **Mapper plugins** (e.g., RegexMapper) update the standardized JSON record with the preprocessed MRI files. The **Demographics Mapper** plugin then appends relevant demographic and clinical fields (e.g., age, sex, diagnosis) to each subject record, thereby producing a unified resource that integrates both anatomical and demographic information.

BrainScape framework’s modular, plugin-based architecture supports plugin swaps via configuration files, eliminating the need to alter the codebase. As a result, researchers can readily include additional plugins supporting specialized pipelines for their unique study requirements. BrainScape’s pipeline workflow and configurable plugins provide a robust, adaptable system for MRI data management.

### 2.3 Experimental setup and performance measurement

We conducted experiments on a local workstation equipped with a 13th Generation Intel Core i9-13900K CPU and an NVIDIA GeForce RTX 4070 Ti GPU (12 GB VRAM). The CPU has 24 physical cores running at a speed of up to 5.8 GHz. The pipeline was tested using Python 3.11 on Ubuntu 22.04. To evaluate the BrainScape framework pipeline, we selected the publicly available VASP dataset (Peelle et al., 2022) from OpenNeuro, which includes T1w and T2w MRI scans for 60 subjects (120 MRI scans in total).

We utilized several Python packages to capture performance metrics: “time” for measuring wall-clock time, resource for computing CPU time, psutil to calculate average CPU utilization, pynvml for GPU usage and memory consumption, and CodeCarbon to estimate electricity consumption (kWh) and ${\text{CO}}_{2}$-equivalents (${\text{CO}}_{2}$eq) emissions, using the carbon intensity data from New Zealand’s local electricity grid.

Currently, the pipeline is designed to process datasets sequentially. Nevertheless, its modular design and configurable workflow support running multiple datasets in parallel, which will be effective in reducing the overall wall-clock time.

### 2.4 Hardware and software requirements

The BrainScape framework is designed to run on standard 64-bit Linux distributions and has been tested on Ubuntu 22.04. Additionally, it supports Windows 10 and 11 through the Windows Subsystem for Linux 2 (WSL2). Essential software prerequisites include Miniconda for managing a dedicated Python (version 3.11) environment and AWS CLI v2 for accessing and downloading MRI data from Amazon S3-hosted repositories, such as OpenNeuro. A GPU is recommended to accelerate the computationally intensive preprocessing task of skull stripping performed by the HD-BET brain-extraction tool (Isensee et al., 2019) in the BraTS plugin. However, a GPU is not mandatory; CPU-only systems can still run the pipeline, though they will take longer to preprocess. Furthermore, BrainScape has been successfully deployed and tested on the New Zealand eScience Infrastructure (NeSI) high-performance computing (HPC) platform, demonstrating its scalability and compatibility with cluster computing environments. NeSI compute nodes run Rocky Linux, a widely used Red Hat-compatible operating system standard across large-scale HPC facilities. Detailed installation instructions, configuration guidelines, and execution documentation are available on the BrainScape GitHub repository.

## 3 Results

### 3.1 Overall dataset composition

The BrainScape dataset includes four anatomical MRI contrasts, that is, T1-weighted (T1w), T2-weighted (T2w), gadolinium-enhanced T1-weighted (T1Gd), and fluid-attenuated inversion recovery (FLAIR). Furthermore, it integrates 160 datasets from diverse open-source repositories and individual research studies. After quality control and visual inspection (e.g., excluding MRIs with artifacts, corrupted and poor-quality scans), 27227 unique subjects remain in the database, yielding 46583 MRI scans overall.

Among these, 31411 are T1w, 7150 are T2w, 1470 are T1Gd, and 6552 are FLAIR. As summarized in Table 1, the distribution of modalities is uneven, with T1w scans representing a larger share of the total MRIs. Yet, the inclusion of T2w and FLAIR scans extends the framework’s utility to applications where multi-contrast data are essential (e.g., lesion analysis, tissue segmentation).

**Table 1.** MRI modality distribution.

Modality	Count	(% of Total)
T1w	31411	67.43
T2w	7150	15.35
T1Gd	1470	3.16
FLAIR	6552	14.07

As shown in Table 1, T1w scans are substantially more common than T2w, T1Gd, and FLAIR scans. Among the 160 datasets included in BrainScape, only 1 dataset (BRATS) provides T1Gd MRI scans. T2w MRI scans are available in 33 datasets, FLAIR scans in 13 datasets, and T1w scans in 160 datasets. However, the number of sessions per subject also varies, ranging from a single scan per subject to multiple scans per subject. The BrainScape dataset retains session information and the original dataset structures, ensuring flexibility for downstream analyses. For complete details of each individual dataset and the available MRI modalities, refer to Supplementary Table S1 in the Supplementary Materials.

Since T1w scans are more common than other modalities, analyses relying primarily on T2w, T1Gd, or FLAIR scans may include fewer subjects. However, the availability of multimodal MRI scans remains valuable for specialized clinical studies or advanced modeling tasks such as lesion segmentation. Researchers can leverage BrainScape’s flexibility to utilize the entire dataset or a subset of the dataset, depending on the specific requirements of the study.

The MRI scans aggregated in the BrainScape dataset originate from scanners with diverse hardware, manufacturers, and magnetic field strengths. Among the 46583 MRIs included, MRI scanner metadata were available for 24508 scans (52.61%), while scanner information for the remaining 22075 scans (47.39%) was missing (see Supplementary Table S2). Furthermore, among the 24508 MRIs with scanner information, 22089 MRIs had magnetic field strength information available. MRI data originated primarily from three major manufacturers (Siemens, Philips, and GE), with Siemens scanners being the most prevalent across all modalities. The dataset predominantly includes 3.0 Tesla (T) MRI scanners (18147 scans), followed by 1.5T (2584 scans), 4.0T (1301 scans), and a small subset from 7.0T scanners (57 scans). The BrainScape dataset contains scans from various manufacturers, models, and field strengths, mirroring the variability seen in clinical and research imaging. This diversity makes the BrainScape dataset an excellent resource for training AI models that are both robust and generalizable. Scanner metadata, including the manufacturer name, model details, and field strength information, are stored in the BrainScape JSON record.

**Fig. 2.**
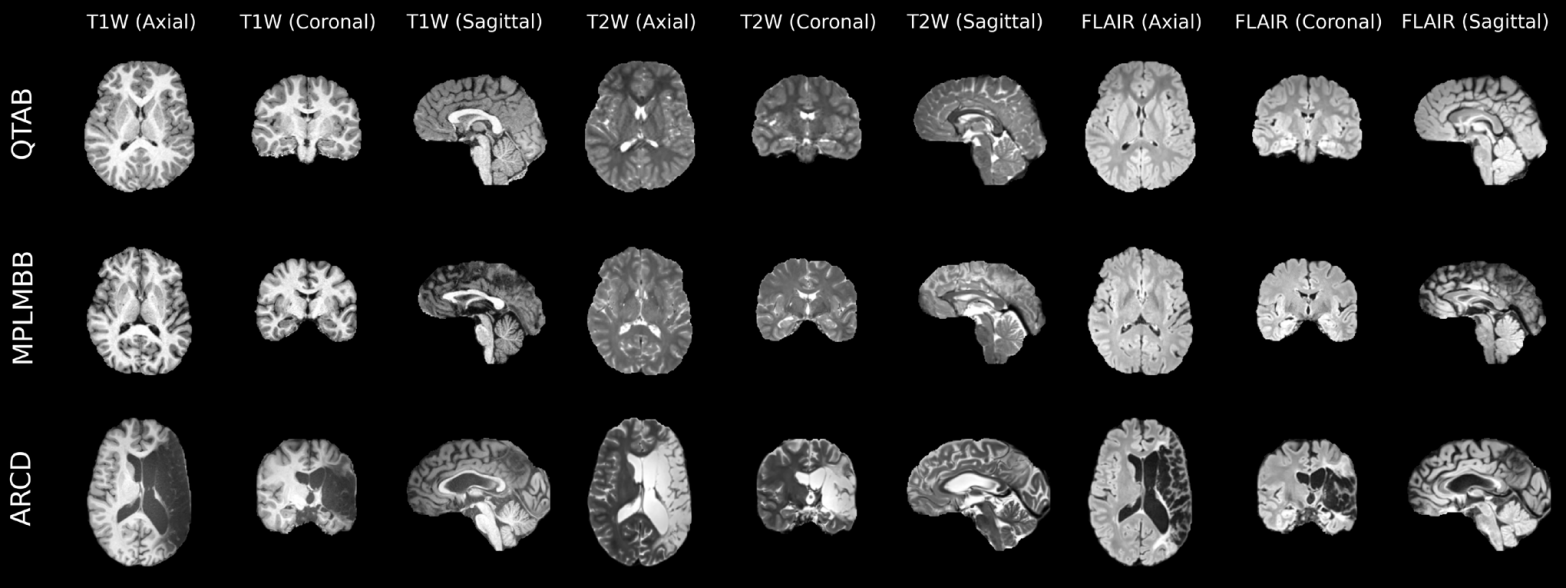
Multimodal MRIs of three subjects from different datasets with each subject having T1w, T2w, and FLAIR modalities. The vertical axis (Y-axis) lists each subject by its source dataset identifiers (detailed in the Supplementary Material), while the horizontal axis shows axial, coronal, and sagittal slices for each modality. Here, the subject from the “QTAB” dataset is a 10-year-old female, the subject from “MPLMBB” is a male aged between 65 and 70 years, and the subject from “ARCD” is a 40-year-old male with a stroke.

Figure 2 illustrates how BrainScape integrates multimodal MRI data from diverse heterogeneous datasets, with varying participant demographics and clinical profiles. Our framework effectively combines these heterogeneous datasets and enables researchers to examine both healthy individuals and those with specific conditions (such as stroke). Moreover, BrainScape preserves the demographic and clinical metadata, allowing researchers to readily filter or subgroup images by attributes such as age, sex, or clinical diagnosis. This flexibility supports a wide range of analyses, from large-scale population studies to more focused clinical investigations.

#### 3.1.1 Demographics information

A total of 27227 subjects were included after inspection from a total of 160 datasets. Of these, 25052 subjects have demographic information, while 2175 subjects lack any recorded demographic details. The demographic data include age, sex, race, handedness, education level, socio-economic status, body mass index, and brain disorders. Table 2 provides a comprehensive overview of the availability of each demographic field across all of the datasets.

**Table 2.** Count of subjects with each demographic field.

Demographic field	Number of subjects
Specific age	20409
Age range	1950
Sex	22526
Handedness	8099
Race	2801
Education	2651
Socio-economic status	1147
Body mass index	2846
Brain disorders	4668
No demographics	2175

It is evident that age (or age range) and sex are among the most crucial and commonly analyzed demographics. According to Table 2, 20409 subjects have an explicit age value, 1950 subjects include a recorded age range label, and 22526 subjects have sex information. Of these, 22138 subjects possess both age (or age range) and sex information.

**Fig. 3.**
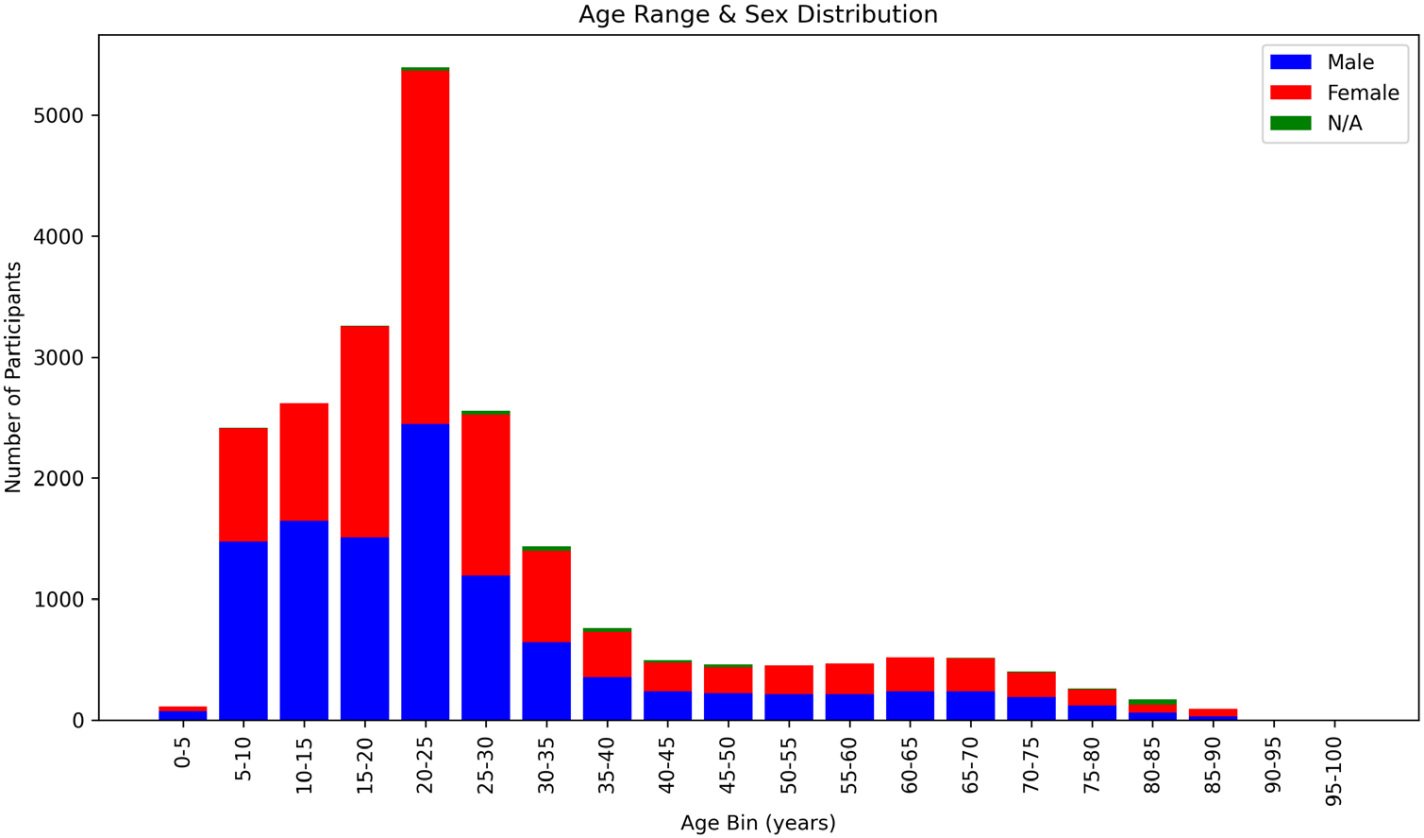
Histogram of Age (or Age Ranges) by Sex. Each bar represents a bin of age or an age range, subdivided by sex categories (male, female, n/a).

Figure 3 presents a histogram illustrating how the dataset’s age distribution is subdivided by sex categories. Specifically, the histogram organizes age (or age range) into bins along the horizontal axis, whereas the vertical bars representing the participants count, color-coded to indicate the sex categories: “male,” “female,” and “n/a.” Note: 388 subjects who have only sex information and lack age or age range information are omitted from the histogram.

Overall, the mean age (excluding entries with only age range data) is 27.09 years (SD = 18.05). By categorizing individual ages into bins, as illustrated in Figure 3, the median age range is determined to be 20–25 years. The sex distribution among subjects with available sex information, regardless of age data, includes 11260 males and 11253 females, totaling 22513 subjects.

Beyond age and sex, additional demographic information such as race, handedness, education level, and socio-economic status (SES) was available only for subsets of participants, with notable variability across datasets (see Table 3). Out of 2801 subjects with available race information, the majority identified as White (1949; 69.58%), followed by Black or African American (476; 16.99%) and Asian (217; 7.75%). Handedness information, available for 8099 subjects, indicated a majority were right-handed (7503; 92.64%), while left-handed (534; 6.59%) and ambidextrous (62; 0.77%) participants were less common.

**Table 3.** Distribution of demographic variables.

Variable	Category/level	Count (n)	Percentage (%)	Total (n)
Race	White	1949	69.58	2801
	Black	476	16.99	
	Asian	217	7.75	
	American Indian/Alaska native	5	0.18	
	Hawaiian/Pacific Islander	5	0.18	
	Two or more races	96	3.43	
	Other	53	1.89	
Handedness	Right (R)	7503	92.64	8099
	Left (L)	534	6.59	
	Ambidextrous (A)	62	0.77	
Education	Low (primary/HS)	331	12.49	2651
	Medium (college)	1008	38.02	
	High (≥ bachelors)	1312	49.49	
Socio-economic status	Low	428	37.31	1147
	Medium	423	36.88	
	High	296	25.81	

Frequencies and percentages of race, handedness, education, and socio-economic status.

Educational information for 2651 subjects was categorized into 3 distinct groups: low, medium, and high. The low education group (331 subjects, 12.49%) includes participants who completed primary education or obtained a high school diploma as their highest education level. The medium education group (1008 subjects, 38.02%) consists of participants who completed some form of post-secondary education below a 4-year degree, such as college, a diploma, or an associate’s degree. The higher education category (1312 subjects, 49.49%) comprises individuals who have completed at least a bachelor’s degree, including master’s or doctoral degrees (PhD). Similarly, socio-economic status (SES), available for 1147 subjects, is categorized into low (428 subjects, 37.31%), medium (423 subjects, 36.88%), and high (296 subjects, 25.81%) groups. Socio-economic status in the BrainScape dataset is derived from four contributing datasets: AOMIC (Snoek et al., 2021), BEANS (Turesky et al., 2021), PAIC (Surani et al., 2024), and SQFC (Miranda-Angulo et al., 2024). In AOMIC, SES is represented on a scale from 2.0 to 6.0 in 0.5 increments, calculated from household income and average parental education; BrainScape maps this scale into Low (2.0–3.0), Medium (3.5–4.5), and High (5.0–6.0). The SQFC dataset provides SES information in three levels (Low, Medium, High), which BrainScape incorporates without modification. All participants in the BEANS and PAIC datasets are classified as Low, as both studies focus on children growing up in extreme poverty.

#### 3.1.2 Participants from clinical cohorts

A total of 4668 participants who are part of the BrainScape dataset have been diagnosed with disorders. These disorders include neurological disorders (stroke, prosopagnosia, dysembryoplastic neuroepithelial tumor (DNT), gliosis, and epilepsy), psychiatric disorders (depression and schizophrenia), and developmental disorders (attention-deficit/hyperactivity disorder (ADHD) and autism spectrum disorder (ASD)). Table 4 lists the number of subjects diagnosed with each disorder among the overall 27227 individuals.

**Table 4.** Number of subjects with specific disorders.

Disorder	Number of subjects^a^
Stroke	215
Acute ischemic stroke	1316
Schizophrenia	119
Depression	120
ADHD	73
ASD	692
Bipolar disorder	49
Prosopagnosia	28
Epilepsy	350
Focal epilepsy	236
Tumor	1470
Gliosis	4
DNT	32
Aneurysm	153

a Only participants who tested Y (Yes) for each disorder are counted here.

Table 4 highlights that 215 participants have been diagnosed with stroke, 350 with epilepsy, and so on. The 236 subjects with focal epilepsy consist of 110 subjects with focal cortical dysplasia and 126 subjects with hippocampal sclerosis. In total, 4668 participants have at least 1 neurological, psychiatric, or developmental disorder.

#### 3.1.3 Quality control and visual inspection

All MRI datasets included in this study underwent visual inspection to identify and exclude scans presenting various artifacts, including motion artifacts, susceptibility distortions, aliasing (wrap-around) artifacts, and Gibbs ringing artifacts. Similarly, subjects with poorly defaced MRIs, where portions of the brain were inadvertently removed, were excluded. For transparency and traceability, any scan failing visual quality checks is listed in the corresponding dataset’s metadata, enabling researchers to audit, revisit, or reverse these exclusions if required.

**Fig. 4.**
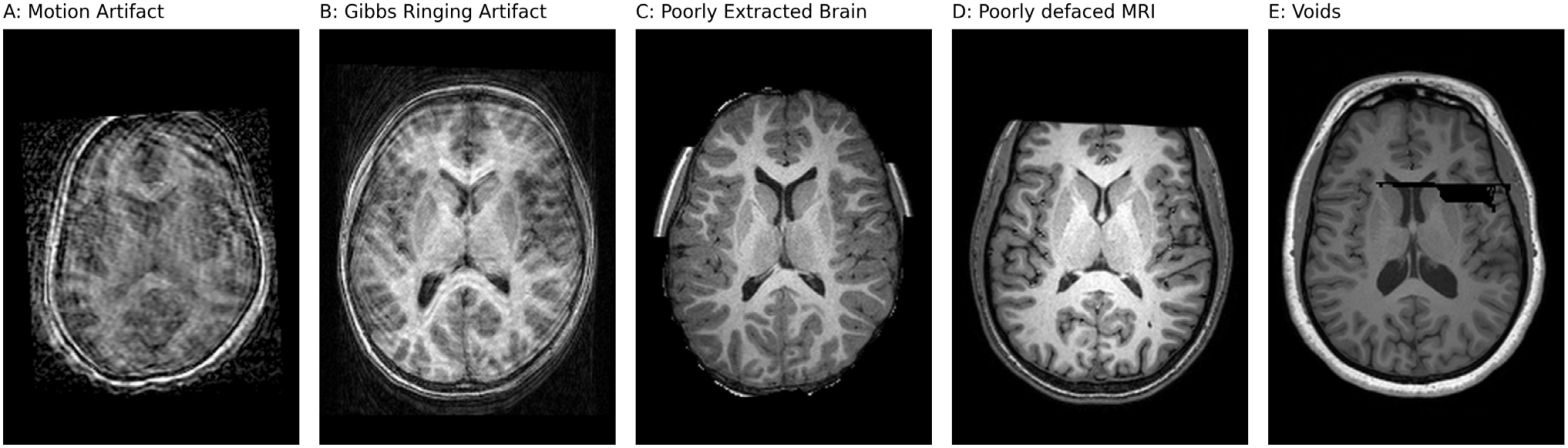
Examples of MRI scans excluded during visual quality inspection: (A) motion artifacts, (B) Gibbs ringing artifact (truncation artifact), (C) poorly extracted brain regions, (D) inadequate defacing with cropped brain regions, and (E) missing or void pixels.

This process has thus far resulted in the removal of 1516 subjects across 81 datasets from the total of 160 included in the repository. Additionally, 5 datasets out of the total 160 have brain MRIs that were already skull stripped. However, we opted to exclude scans with incomplete skull stripping, such as those illustrated in Figure 4. Reprocessing datasets that contain both fully and partially skull-stripped MRIs with the default BraTS preprocessing plugin, which uses HD-BET brain-extraction tool (Isensee et al., 2019), results in over-erosion of brain tissue during brain extraction. We decided to drop incomplete skull-stripped scans from such datasets to keep a single default BraTS preprocessing pipeline for the BrainScape dataset release, and because this issue affected only a small subset of subjects (approximately 130 subjects). Figure 4 illustrates examples of the excluded MRI scans, highlighting the types of artifacts and issues that led to their removal. By making the exclusion criteria explicit and open to re-evaluation by the neuroscience community, we reduce the risk of perpetuating errors and encourage collaborative quality assurance, ultimately enhancing the overall integrity and reproducibility of the dataset.

### 3.2 Computational performance and carbon footprint

The BrainScape framework completed the full workflow for the VASP dataset (120 MRI scans) in approximately 33.3 minutes of wall-clock time. The recorded CPU utilization was only 21.85%, reflecting the predominantly sequential structure of our current implementation. In total, the pipeline took 228 minutes of CPU time across the 24 CPU cores, indicating further room for parallelization. Future updates to BrainScape are expected to enable running multiple datasets concurrently, which can significantly shorten wall-clock time and increase overall efficiency. Real-time monitoring of GPU resources revealed that usage peaked during skull stripping (during preprocessing), with memory consumption reaching about 5406 MB (44% of the available 12 GB). Outside of these intensive operations, the GPU remained largely idle, resulting in an average utilization of 24.78% throughout the entire workflow. Incorporating additional datasets is expected to proportionally increase wall-clock time and CPU time.

We also evaluated the pipeline’s environmental impact by considering local grid emission factors in New Zealand. Over the entire run on the VASP dataset, the pipeline consumed an estimated 0.07065 kilowatt-hours (kWh) of electricity, corresponding to 0.00796 kg of CO_2_eq emissions. The 0.07065 kWh is roughly the energy needed to keep a 10 W LED lamp on for 7 hours, or to run a typical 1.5 kW space heater for a little under 3 minutes. As more datasets are included, the energy consumption and carbon footprint may rise proportionally; however, workflow parallelization can help maintain reasonable total run times and minimize ecological impact. By documenting these performance metrics, we highlight the computational overhead and the opportunity to reduce processing times and emissions through concurrent dataset handling. We anticipate that implementing parallel dataset processing pipelines will improve BrainScape’s computational performance and carbon footprint.

## 4 Discussion

In this paper, we introduce BrainScape, an easy-to-use resource that combines an open-source, plugin-based software framework with a collated dataset distributed as a curated set of dataset-specific configuration files. BrainScape simplifies the downloading, management, aggregation, and preprocessing of publicly available anatomical MRI data, making it straightforward for researchers to regenerate standardized, analysis-ready datasets. BrainScape addresses known challenges associated with aggregating multimodal MRI datasets, including the fragmentation of specialized clinical data, incompatible dataset formats and organization, lack of automated data-handling workflows, and inconsistencies in demographic and clinical metadata. BrainScape integrates heterogeneous MRI datasets while preserving each dataset’s original structure, subject organization, and licensing details. This design choice minimizes the risks of data duplication, data loss, and inadvertent bias, enabling researchers to easily and reliably merge data from multiple sources for more comprehensive analyses.

One of the strengths of BrainScape is its flexibility and scalability. The configurable and modular plugin-based architecture of the BrainScape framework enables seamless integration of new data sources, imaging modalities, and preprocessing pipelines without requiring any modifications to the core codebase. Researchers can add new multimodal MRI datasets by including new configuration files. Additionally, the BrainScape framework provides the flexibility to include plugins for downloading data from other open-source MRI databases and specialized preprocessing plugins for specific studies that may consist of custom preprocessing pipelines. This design facilitates an environment of continuous improvement and customization. We integrate 160 diverse datasets, demonstrating the framework’s scalability and capability to handle large-scale data aggregation. Furthermore, BrainScape’s ability to trace each file back to its source and maintain dataset-specific configurations demonstrates reproducibility by ensuring that the inclusion criteria, quality control outcomes, and plugin parameters are recorded in a transparent and standardized manner.

By automating data downloading, mapping, validation, and preprocessing, BrainScape framework reduces the manual labor required for large-scale, multi-site data pooling. Researchers can, therefore, shift their focus away from tedious preparation tasks and concentrate on analysis, interpretation, and theory development. This is particularly helpful for deep learning and machine learning applications, where model performance benefits from the diversity and volume of training data (Dishner et al., 2024). Beyond aggregating diverse MRI scans, BrainScape also supports attaching demographic fields (such as age, sex, handedness, and clinical status), enabling researchers to build inclusive datasets that capture control and patient cohorts. This diversity is particularly crucial for rare phenotypes and targeted clinical cohorts, which may be overlooked in large-scale aggregated datasets, yet offer critical insights into neurological function and pathology (P. M. Thompson et al., 2014).

The BrainScape framework is designed to interoperate seamlessly with the broader neuroinformatics ecosystem, complementing existing tools rather than seeking to replace them. At the level of data organization, BrainScape adopts the Brain Imaging Data Structure (BIDS), preserving each dataset’s native subject, session, and modality hierarchy. Each file is systematically mapped to a JSON record containing subject, session, modality, and demographic information. The BrainScape framework also integrates with containerized plugin implementations. For example, its sMRIPrep plugin retrieves and runs the official nipreps/smriprep Docker image, with the resulting preprocessed MRI data subsequently remapped to their corresponding JSON records through the RegexMapper. The framework also goes well with the open neuroimaging repositories The currently available Downloader plugins automate downloads from resources such as OpenNeuro, the Human Connectome Project’s Amazon S3 data bucket, and Synapse.

BrainScape framework does not implement data harmonization within its pipeline to reduce variability across imaging sites. Our primary objective was to preserve the natural heterogeneity and variability across diverse MRI datasets to support downstream deep learning applications, and to allow users to implement their own harmonization approaches. Harmonization often reduces real-world variability critical for training robust and generalizable AI models (Adkinson et al., 2024). Therefore, we decided to exclude the harmonization step to preserve each dataset’s original characteristics.

A key motivation for pooling diverse datasets is to improve generalizability, bridging the gap between replicable findings and real-world clinical applicability (Adkinson et al., 2024; Marek & Laumann, 2024; Yang et al., 2024). Although many large-scale consortia provide extensive datasets that support reproducibility, they often exhibit limited demographic representation and constrained dataset variability, reducing their broader generalizability. Such constraints can lead to shortcut learning in machine learning models, whereby models inadvertently capture patterns associated with confounding variables rather than true brain–behavior relationships (Marek & Laumann, 2024; Yang et al., 2024). Another motivation behind BrainScape development is the recognition that numerous specialized MRI datasets likely remain underutilized compared with the widely used large-scale consortia datasets. Although these datasets often contain valuable information on rare phenotypes and specific clinical conditions, they are underutilized in pooling efforts due to complexities in data aggregation. By systematically integrating these diverse datasets, BrainScape promotes dataset heterogeneity, facilitating the development of robust, transferable models that accurately reflect the true variability observed in both healthy and clinical populations.

Despite its strengths, the collated BrainScape dataset shares some limitations common to the field. First, there is an uneven coverage of demographic and clinical metadata across datasets, which may constrain the generalizability of analyses and introduce sampling biases. This fragmented demographic information likely arises because the BrainScape dataset includes resources from individual researchers with varying funding levels and research targets. As a result, we cannot expect the same level of annotations as found in multi-million dollar projects such as HCP and UK Biobank (Miller et al., 2016; Van Essen et al., 2013). Furthermore, the distribution of demographic variables does not fully reflect real-world populations. For instance, the current age distribution skews toward individuals between 5 and 30 years, with fewer participants above 30 years. Similarly, most participants self-identify as White, while other racial or ethnic groups are comparatively underrepresented. Educational levels also exhibit an imbalance, with more than half of the participants reporting high-level education (bachelor’s, master’s, or PhD). Although these demographic imbalances mean the integrated dataset may not yet comprehensively represent the broader population. We aim to progressively reduce these limitations by incorporating more diverse, heterogeneous repositories into the BrainScape dataset. Potential reasons for the current bias include the nature of volunteer-based studies, recruitment from university populations, and missing demographic fields across datasets. Ongoing integration of additional datasets, particularly those emphasizing broader demographic and clinical diversity, will further enhance generalizability.

Second, the distribution of MRI modalities is skewed, with a relatively high number of T1w images than T2w and FLAIR scans. This imbalance might limit the scope of studies that require multimodal MRI data for effective analysis, such as automated lesion segmentation (Menze et al., 2014; Spitzer et al., 2022). Over time, community-driven contributions should address this imbalance by integrating additional datasets containing richer multimodal MRIs. Furthermore, licensing restrictions remain a concern; reliance on publicly available data means that the framework is subject to existing licensing restrictions and access barriers inherent in some proprietary datasets. Although BrainScape tracks licensing terms and usage permissions, researchers must be aware of data use agreements and privacy regulations to ensure responsible collaboration. Additionally, downloading and preprocessing large-scale MRI data require significant time and computational resources, potentially extending the overall study timeline and requiring access to high-performance computing resources.

BrainScape dataset currently includes 160 diverse brain MRI datasets and will continue to grow as new data are added. Although no predefined schedule exists for identifying and integrating additional datasets, we actively manage the repository and incorporate new open-source datasets. We also hope future upgrades will come through community collaboration on the BrainScape GitHub repository, which includes detailed tutorials for developing new plugins and integrating additional datasets.

Looking ahead, BrainScape can be extended to incorporate diffusion-weighted imaging (DWI), resting-state fMRI, and other specialized MRI sequences, enhancing the dataset’s capability to support multimodal analyses of structural and functional connectivity (Van Essen et al., 2013). Furthermore, including an automated quality control module into the pipeline, such as machine learning-based artifact detection, makes it easier to include additional open-source datasets by reducing the need for manual quality control, which is labor intensive and time consuming. Such a module may also automatically detect poor-quality MRIs overlooked during manual review. Another key improvement is parallelizing the workflow; currently, the BrainScape framework processes datasets sequentially, but enabling concurrent dataset handling could significantly reduce the overall processing time. Furthermore, the BrainScape framework can be enhanced with new preprocessing plugins designed to implement specialized pipelines for targeted research studies. Meanwhile, the BrainScape dataset can also be enhanced by integrating new open-source brain datasets targeted at specialized analyses and rare phenotypes.

In conclusion, BrainScape addresses bottlenecks in MRI data aggregation and preprocessing by collecting datasets of diverse origins, automating workflows, and preserving original dataset integrity. It will serve as an effective tool for the neuroscience community by enabling the creation of more comprehensive, representative, and robust datasets. While challenges such as inconsistent demographic data and licensing restrictions persist, the BrainScape flexible and transparent design allows for ongoing improvements through active community collaboration. We believe that BrainScape contributes positively to open science, to enhance our understanding of the brain.

## Supplementary Material

Supplementary Material

## Data Availability

All source code, default plugins, and dataset-specific configuration files required to reproduce the BrainScape workflow are openly available under the MIT licence at https://github.com/yasinzaii/BrainScape. Since BrainScape automatically integrates publicly available MRI data from multiple open repositories, no derivatives (preprocessed dataset) are distributed. Instead, any researcher can locally regenerate the same derivatives by cloning the repository, and executing the workflow with the provided configuration files. This approach avoids storage overhead of hosting duplicate derivatives and preserves full provenance, since the choice of plugins and their parameters is recorded in every configuration file. For detailed usage instructions, refer to the README file or contact the authors for further clarification. Comprehensive user documentation is hosted alongside the code in the BrainScape GitHub repository. The root “README.md” file provides a concise overview of the framework, outlines prerequisites, offers quick-start and configuration guides, and explains how to include additional datasets. In addition, a dedicated tutorials directory contains detailed guides that show how to add a new dataset, attach or extend demographic metadata, follow an end-to-end workflow walkthrough, explore the BrainScape framework API, and develop custom downloader or preprocessing plugins. These documents are updated frequently, ensuring readers can access the latest information and implementation details.
