# Supplementary material for: BrainScape: An open-source framework for integrating and preprocessing anatomical MRI datasets
Source: Imaging Neurosci (Camb). 2025 Oct 22;3:IMAG.a.944. doi: 10.1162/IMAG.a.944 (PMC12547439; doi:10.1162/IMAG.a.944)
Supplement: Supplementary Material [file IMAG.a.944_supp.pdf]

BrainScape: An Open-Source Framework for Integrating and Preprocessing Anatomical MRI Datasets

Muhammad Nabi Yasinzai,[1] Remika Mito,[2] Mangor Pedersen[1*]

[1]Department of Psychology & Neuroscience, Auckland University of Technology, Auckland, New Zealand.

[2]Department of Psychiatry, University of Melbourne, Melbourne, Victoria 3053, Australia.

*Correspondence: mangor.pedersen@aut.ac.nz

September 12, 2025

1 Supplementary Material

1.1 Overview of Datasets Included in BrainScape

We provide a detailed table summarizing essential metadata for each dataset included in BrainScape. Supplementary Table S1 provides information on the 160 MRI studies and datasets currently included in the BrainScape dataset. As the project evolves, additional datasets will be added, further expanding this list. The following section describes the columns of the table.

1. **Identifier**: Each dataset is assigned a unique identifier (e.g., "AOMIC", "QTAB") to facilitate referencing within BrainScape. All MRI scans and associated metadata for each dataset are organized in dedicated folders named after their identifiers, ensuring traceability to the original source or study. Hyperlinks embedded in the identifier column provide direct access to the corresponding source dataset repository. Some datasets may require authorization or the completion of specific user agreements for access.

2. **Dataset Name**: This column lists the name of the dataset or study from which the anatomical

MRI scans were obtained. When applicable, a citation is included to acknowledge the contributions of the original dataset creators.

3. **License**: This column specifies the licensing terms under which each dataset is made available. Many datasets obtained through OpenNeuro (Markiewicz et al., 2021) are distributed under a CC0 license, allowing unrestricted reuse. However, some data sources (e.g., ABIDE (Di Martino et al., 2014), ABIDE2 (Di Martino et al., 2017), ADHD200 (consortium, 2012), CORR (Zuo et al., 2014), CMIHBN (Alexander et al., 2017), INDINKI (Nooner et al., 2012)) are governed by CC-BY-NC or similarly restrictive licenses. These datasets often require a signed user agreement, an approved access application, or both. Users are responsible for reviewing and complying with all applicable licensing conditions before using these datasets.

4. **Subjects**: This column reports the number of participants whose anatomical scans are included in this study. In some instances, this total may be lower than the number of participants in the original dataset because scans that failed visual quality inspections were excluded. Records of these excluded subjects are maintained in the corresponding dataset metadata, allowing researchers to re-evaluate or reverse exclusion decisions if necessary.

5. **T1w, T2w, and FLAIR**: These columns display the number of scans available for each MRI modality in every dataset. Individual subjects may undergo multiple scanning sessions, which can result in scan counts exceeding the total number of subjects.

Note: gadolinium-enhanced T1-weighted (T1Gd) MRIs are only provided by the BRATS dataset, therefore this modality is not included in Supplementary Table S1.

Supplementary Table S1: Comprehensive List of Integrated Anatomical MRI Datasets

Identifier	Dataset Name	License	Subjects	T1w	T2w	Flair
ABIDE	Autism Brain Imaging Data Exchange I - ABIDE I (Di Martino et al., 2014)	CC BY-NC-SA 3.0	1034	1034	0	0
ABIDE2	Autism Brain Imaging Data Exchange II - ABIDE II (Di Martino et al., 2017)	CC BY-NC-SA 3.0	588	588	0	49

Continued on next page

Supplementary Table S1 – continued from previous page

Identifier	Dataset Name	License	Subjects	T1w	T2w	Flair
ADHD200	ADHD-200 (consortium, 2012)	CC BY-NC 4.0	906	906	0	0
AHDC	Structural and functional MRI dataset from the Adolescent Health and Development in Context (AHDC) study (Boettner et al., 2019)	CC0	262	262	257	0
AHRBS	Adolescent Health Risk Behavior Study: Monetary Incentive Delay (MID) task data (Demidenko et al., 2024)	CC0	60	120	0	0
AOMIC	Amsterdam Open MRI Collection (AOMIC) - AOMIC-ID1000 (Snoek et al., 2021)	CC0	926	2762	0	0
APTSIE	Agreeableness personality trait and social information encoding (Arbula et al., 2021)	CC0	55	55	0	0
ARCD	Aphasia Recovery Cohort (ARC) Dataset (Gibson et al., 2024)	CC0	215	419	412	221
ARDACA	Age-related differences in auditory cortex activity during spoken word recognition (Rogers et al., 2020)	CC0	61	61	61	0
ATTEXP	AttExp_fMRI (Penalver et al., 2024)	CC0	52	52	52	0
BATB	Bilingualism and the brain (DeLuca et al., 2019)	CC0	64	64	0	0
BCMD	Brain Correlates of Math Development (Suárez-Pellicioni et al., 2019)	CC0	126	186	0	0
BCPPR	Brain connectivity predicts placebo response across chronic pain clinical trials (Tétreault et al., 2016)	CC0	74	74	0	0
BCSP	Brain correlates of speech perception in schizophrenia patients with and without auditory hallucinations (Soler-Vidal et al., 2022)	CC0	71	71	0	0
BDDR	Brain Development of Deductive Reasoning (Lytle et al., 2020)	CC0	53	53	0	0
BEANS	BrainMorphometry DiminishedGrowth BEAN study 2021 (Turesky et al., 2021)	CC0	71	71	0	0
BMDMS	Brain mechanisms disciminating enactive mental simulations of runnning and plogging (Philips et al., 2024)	CC0	90	90	0	0
BRATS	Brain Tumor Segmentation (BraTS) Adult Glioma 2023 Challenge Dataset (Baid et al., 2021)	CC0	1470	1470	1470	1470

Continued on next page

Supplementary Table S1 – continued from previous page

Identifier	Dataset Name	License	Subjects	T1w	T2w	Flair
CCCS	Caltech Conte Center - A multimodal data resource for exploring social cognition and decision-making. (Kliemann et al., 2022)	CC0	117	249	73	0
CCNFT	The role of superstition of cognitive control during neurofeedback training - part 1 (Grössinger et al., 2021)	CC0	53	53	53	0
CFME	CAT Faces MRI experiment	CC0	50	76	0	0
CHLStudy	Characterizing habit learning in the human brain at the individual and group levels: a multi-modal MRI study (Gera et al., 2023)	CC0	123	223	0	0
CICT	Cue Induced Craving task Dataset (Tomova et al., 2020)	CC0	96	96	96	0
CLLD		CC0	51	51	0	0
CMDD	Cortical myelin measured by the T1w/T2w ratio in individuals with depressive disorders and healthy controls (Baranger et al., 2021)	CC0	87	88	88	0
CMIHBN	CMI Healthy Brain Network (Alexander et al., 2017)	CC BY 4.0	1654	1389	319	725
CORR	Consortium for Reliability and Reproducibility (CoRR) (Zuo et al., 2014)	CC BY 4.0	1346	1478	0	0
CPRO	Concrete Permuted Rule Operations (Ito et al., 2017)	CC0	94	94	94	0
CRND	Cerebrovascular Reactivity Normative Dataset (Rovai et al., 2024)	CC0	50	50	0	0
CSMLP	Cross-Sectional Multidomain Lexical Processing (Lytle et al., 2020)	CC0	73	73	0	0
CSNPS	Cross-stage neural pattern similarity in the hippocampus predicts false memory derived from post-event inaccurate information (Shao et al., 2023)	CC0	55	55	0	0
CTM	Cognitive Control Theoretic Mechanisms of Real-time fMRI-Guided Neuromodulation (CTM) (Bush et al., 2022)	CC0	73	73	0	0
CTS	Cognitive Training (Kable et al., 2017)	CC0	157	278	275	0

Continued on next page

Supplementary Table S1 – continued from previous page

Identifier	Dataset Name	License	Subjects	T1w	T2w	Flair
CTStudy	Cognitive tasks, anatomical MRI, and functional MRI data evaluating the construct of self-regulation (Bissett et al., 2024)	CC0	107	179	138	0
CWHSH	Can we have a second helping? A replication study on the neurobiological mechanisms underlying self-control (Scholz et al., 2022)	CC0	80	80	0	0
DCPC	Developmental change in prefrontal cortex recruitment supports the emergence of value-guided memory (Nussenbaum and Hartley, 2021)	CC0	90	93	88	0
DFN	Differentiation of functional networks during long-term memory retrieval in children and adolescents (Fynes-Clinton et al., 2019)	CC0	60	60	0	0
DLBStudy	The Dallas Lifespan Brain Study (McDonough et al., 2016)	CC0	393	800	0	805
DNRNP	Deciphering the neural responses to a naturalistic persuasive message (Ntoumanis et al., 2024)	CC0	50	50	0	0
DPCASL	DP-pCASL data (CBF, ATT, BBB kw) from 186 cognitively normal participants (8-92 years) (Shao et al., 2024)	CC0	49	49	0	0
DPTStudy	Dynamic Passive Threat (Meyer et al., 2019)	CC0	72	72	72	0
DSNP	Development of Symbolic Number Processing	CC0	55	55	0	0
ECStudy	The Energetic Costs of the Human Connectome (Castrillon et al., 2023)	CC0	19	28	0	0
EDBP	Emotion and Development Branch Phenotyping and DTI (2012-2017) (McKay et al., 2024)	CC0	129	129	130	0
EEAR	Examining effects of arousal on responses to salient and non-salient stimuli in younger and older adults (Lee et al., 2018)	CC0	49	49	0	0
EMMBC	Evaluating methods for measuring background connectivity in slow event-related fMRI designs (Frank and Zeithamova, 2023)	CC0	56	56	0	0
ERAB	Emotion regulation in the Ageing Brain, University of Reading, BBSRC (Lloyd et al., 2021)	CC0	78	78	0	0

Continued on next page

Supplementary Table S1 – continued from previous page

Identifier	Dataset Name	License	Subjects	T1w	T2w	Flair
ERFF	A behavioral, clinical and brain imaging dataset with focus on emotion regulation of females with fibromyalgia (Balducci et al., 2022)	CC0	66	66	66	0
FBS	Food and Brain Study (Keller et al., 2023)	CC0	78	78	0	0
FC1000	The 1000 Functional Connectomes Project (Biswal et al., 2010)	CC BY-NC 4.0	861	861	0	0
FDNPS	NarrativePuzzle	CC0	65	65	0	0
GOTD	Game of Thrones - A naturalistic viewing dataset (Noad et al., 2024)	CC0	73	73	0	0
HBM	Kwok et al., 2023, Human Brain Mapping (Kwok et al., 2023)	CC0	66	66	0	0
HCP1200	Human Connectome Project 1200 Subjects Data Release (Van Essen et al., 2013)	-	1113	1113	1113	0
HLC	Hearing loss Connectome (Ponticorvo et al., 2022)	CC0	82	82	0	0
HRVStudy	Heart rate variability biofeedback training and emotion regulation (Min et al., 2022)	CC0	171	315	0	0
HSNP	Handedness and Symbolic Number Representation (Goffin et al., 2019)	CC0	53	53	0	0
IBRGU	Increased brain reactivity to gambling unavailability is a marker of problem gambling (Brevers et al., 2021)	CC0	65	65	0	0
IDAS	Interoception during aging: The heartbeat detection task (Dobrushina, Arina, et al., 2020)	CC0	39	39	0	0
IDEAS	The Imaging Database for Epilepsy And Surgery (IDEAS) (Taylor et al., 2024)	CC0	365	365	0	317
IDFPH	Individual differences in frontoparietal plasticity in humans (Boroshok et al., 2022)	CC0	92	92	92	0
IDMA	fMRI Investigations of Individual Differences on Memory Activation for Faces and Words: The Effects of Handedness and Phenomenal Experience (Schmidt and Kirwan, 2024)	CC0	98	98	0	0
IEFA	Isometric exercise facilitates attention to salient events in women via the noradrenergic system (Mather et al., 2020)	CC0	91	91	0	0

Continued on next page

Supplementary Table S1 – continued from previous page

Identifier	Dataset Name	License	Subjects	T1w	T2w	Flair
IHNC	The impact of handedness on the neural correlates during kinesthetic motor imagery: a FMRI study (Crotti et al., 2022)	CC0	51	51	0	0
ILCCB	An fMRI dataset for investigating language control and cognitive control in bilinguals	CC0	75	75	0	0
INDINKI	INDI NKI/Rockland Sample (Nooner et al., 2012)	Attribution Non-Commercial	201	202	0	0
IRAS	Individual risk attitudes arise from noise in neurocognitive magnitude representations. (Garcia et al., 2022)	CC0	64	64	0	0
IRMS	Interacting representations of mental states and traits	CC0	53	53	0	0
IXI	IXI (Information eXtraction from Images) dataset	CC BY-SA 3.0	401	400	397	0
LBCMLP	Longitudinal Brain Correlates of Multisensory Lexical Processing in Children (Lytle et al., 2019)	CC0	184	233	0	0
LLAD	Language Learning Aptitude dataset (Novén et al., 2021)	CC0	57	57	0	0
LPPHK	Le Petit Prince Hong Kong - Naturalistic fMRI and EEG dataset from older Cantonese speakers (Momenian et al., 2024)	CC0	50	50	0	0
LPPStudy	Le Petit Prince - A multilingual fMRI corpus using ecological stimuli (J. Li et al., 2022)	CC0	93	93	0	0
LRES	Learning rules of engagement for social exchange within and between groups (Rojek-Giffin et al., 2023)	CC0	50	50	0	0
LSFD	Large-scale fMRI dataset for the design of motor-based Brain-Computer Interfaces (Bom et al., 2024)	CC0	137	137	0	0
LTMAC	Lausanne_TOF-MRA_Aneurysm_Cohort (Di Noto et al., 2023)	CC0	278	290	0	0
LTS	Listening task (Rogers et al., 2023)	CC0	78	78	78	0
MAICY	Multivariate Assessment of Inhibitory Control in Youth: Links with Psychopathology and Brain Function Dataset (Cardinale et al., 2024)	CC0	118	118	0	0

Continued on next page

Supplementary Table S1 – continued from previous page

Identifier	Dataset Name	License	Subjects	T1w	T2w	Flair
MBMP	Modality-based Multitasking and Practice - fMRI (Mueckstein et al., 2024)	CC0	57	57	0	0
MBSR	Modality-based Multitasking and Practice - fMRI (Seminowicz et al., 2020)	CC0	146	344	0	0
MCAC	Maturational Changes in Anterior Cingulate and Frontoparietal Recruitment Support the Development of Error Processing and Inhibitory Control (Antistate) (Velanova et al., 2008)	CC0	70	70	0	0
MCS	Mapping the Connectome of Synaesthesia - An Open Access MRI Dataset (Racey et al., 2023)	CC0	127	127	127	0
MCStudy	magic_carpet (Feher da Silva et al., 2023)	CC0	94	94	0	0
MEC	Multi-echo Cambridge (Power et al., 2018)	CC0	89	89	0	0
MESD	Multi-echo simultaneous multislice fMRI dataset: Effect of acquisition parameters on fMRI data	CC0	50	50	0	0
MIMR	Multiple interactive memory representations underlie the induction of false memory	CC0	59	59	0	0
MLStudy	Michigan Longitudinal Study (Yau et al., 2012)	CC0	72	150	0	0
MMC	Magic, Memory, and Curiosity (MMC) fMRI Dataset (Ozono et al., 2021)	CC0	50	50	0	0
MPLMBB	The MPI-Leipzig Mind-Brain-Body dataset (Babayan et al., 2019)	CC0	318	319	226	306
MRART	Movement-related artefacts (MR-ART) dataset (Nárai et al., 2022)	CC0	148	148	0	0
MVD	Milky-Vodka	CC0	54	54	0	0
NARPS	NARPS (Botvinik-Nezer et al., 2019)	CC0	108	108	0	0
NARR	Narratives	CC0	334	365	0	0
NCAS	Neurocognitive aging data release with behavioral, structural, and multi-echo functional MRI measures (Setton et al., 2023)	CC0	257	257	0	207
NCStudy	neuroCOVID MRI dWI and fMRI with reversal learning	CC0	100	100	100	0
NDStudy	Functional magnetic resonance imaging data for the neural dynamics underlying the acquisition of distinct auditory categories (Feng et al., 2021)	CC0	55	55	0	0

Continued on next page

Supplementary Table S1 – continued from previous page

Identifier	Dataset Name	License	Subjects	T1w	T2w	Flair
NENST	Neuroimaging evidence for network sampling theory of human intelligence (Soreq et al., 2021)	CC0	60	60	0	0
NES	NeuroEngage	CC0	51	51	0	0
NIMHC	NIMH-CompyPsych MMI (Keren et al., 2021)	CC0	51	51	0	0
NIMHIHV	The NIMH intramural healthy volunteer dataset (Nugent et al., 2022)	CC0	247	249	247	241
NND	Naturalistic Neuroimaging Database (Aliko et al., 2020)	CC0	86	86	0	0
NPCHA	Neuroimaging predictors of creativity in healthy adults (Sunavsky and Poppenk, 2020)	CC0	66	66	66	0
OIAStudy	Odour-imagery ability is linked to food craving, intake, and adiposity change in humans (Perszyk et al., 2023)	CC0	46	46	0	0
PAIC	Psychosocial adversity and inhibitory control: an fMRI study of children growing up in extreme poverty (Surani et al., 2024)	CC0	64	64	0	0
PASQP	Parallel Adaptation of Symbols, Quantities, and Physical Size (Sokolowski et al., 2021)	CC0	52	52	0	0
PDFCC	Parkinson's disease, functional connectivity, and cognition (Wylie et al., 2023)	CC0	57	57	0	0
PDMTA	Probability Decision-making Task with ambiguity (Valdebenito-Oyarzo et al., 2024)	CC0	52	52	52	0
PENAStudy	A naturalistic paradigm to investigate post-encoding neural activation patterns in relation to subsequent voluntary and intrusive recall of distressing events (Visser et al., 2022)	CC0	35	35	0	0
PEPP	Magnetoencephalographic (MEG) Pitch and Duration Mismatch Negativity (MMN) in First-Episode Psychosis (López-Caballero et al., 2024)	CC0	60	60	0	0
PKIK	PenaltyKik.02 (McDonald et al., 2019)	CC0	69	69	0	0
PLS	Pragmatic Language (Reyes-Aguilar et al., 2023)	CC0	144	144	0	0
PMM	Political Moralization (PMM) (Cohen et al., 2024)	CC0	50	50	0	0
POLEX	POLEX	CC0	58	58	0	0
PPS	Paingen_placebo (Botvinik-Nezer et al., 2024)	CC0	394	396	0	0

Continued on next page

Supplementary Table S1 – continued from previous page

Identifier	Dataset Name	License	Subjects	T1w	T2w	Flair
PSED	An open presurgery MRI dataset of people with epilepsy and focal cortical dysplasia type II (Schuch et al., 2023)	CC0	168	168	0	168
PTStudy	The heterogeneity in retrieved relations between the personality trait 'Harm avoidance' and gray matter volumes due to variations in the VBM and ROI labeling processing settings (Van Schuerbeek et al., 2016)	CC0	95	95	0	0
QTAB	Queensland Twin Adolescent Brain (QTAB) (Strike et al., 2023)	CC0	274	480	441	449
QTIM	Queensland Twin IMaging (QTIM) (Strike et al., 2023)	CC0	1167	1301	0	0
RBPL1	The Reading Brain Project L1 Adults (P. Li and Clariana, 2019)	CC0	52	52	0	0
RBPL2	The Reading Brain Project L2 Adults (P. Li and Clariana, 2019)	CC0	56	56	0	0
RRBD	Relationship between resting state functional connectivity and reading-related behavioural measures in 69 adults (Bathelt et al., 2024)	CC0	71	136	0	0
RRFAC	Reading-related functional activity in children with isolated spelling deficits and dyslexia (Banfi et al., 2021)	CC0	58	58	0	0
RSCEP	Resting state with closed eyes for patients with depression and healthy participants (Bezmaternykh et al., 2021)	CC0	70	70	0	0
RSD	Resting-state for 34 younger and 28 older adults (Wahlheim et al., 2021)	CC0	58	58	0	0
RSNA	Exploring the Resting State Neural Activity of Monolinguals and Late and Early Bilinguals (Gold, 2018)	CC0	92	92	0	0
RSPHA	Resting State Perfusion in Healthy Aging (Vidorreta et al., 2013)	CC0	60	60	0	0
RSSE	rsfMRI_single_session_EEG_NF (Dobrushina, Vlasova, et al., 2020)	CC0	51	102	0	0

Continued on next page

Supplementary Table S1 – continued from previous page

Identifier	Dataset Name	License	Subjects	T1w	T2w	Flair
SDIOA	Single Dose Intranasal Oxytocin Administration: Data from Healthy Younger and Older Adults (Liu et al., 2022)	CC0	85	85	0	0
SDRR	In silico discovery of representational relationships across visual cortex (Gifford et al., 2024)	CC0	6	12	0	0
SDSStudy	Structural (t1) images of 136 young healthy adults (Zareba et al., 2022)	CC0	134	134	0	0
SDStudy	Speech disfluencies: Neurophysiological aspect in normal population	CC0	81	81	0	0
SEND	Stanford Emotional Narratives fMRI Dataset	CC0	99	99	0	0
SIVBM	https://openneuro.org/datasets/ds000222/versions/1.0.1 (FitzGerald et al., 2017)	CC0	79	79	0	0
SKIP	SoCal Kinesia and Incentivization for Parkinson's Disease (SKIP): Active Escape (Dundon et al., 2024)	CC0	68	68	0	0
SNRP	An fMRI dataset of social and nonsocial reward processing in young adults (Smith, Wyngaarden, et al., 2024)	CC0	58	58	0	0
SOOP	Stroke Outcome Optimization Project (SOOP) (Absher et al., 2024)	CC0	1493	1493	0	1493
SPCC	Single-pulse TMS fMRI (Glick et al., 2024)	CC0	147	159	0	0
SQFC	Sympathovagal quotient and functional connectivity of control networks are related to gut Ruminococcaceae abundance in healthy men (Miranda-Angulo et al., 2024)	CC0	86	86	0	0
SRPDM	An fMRI Dataset on Social Reward Processing and Decision Making in Younger and Older Adults (Miranda-Angulo et al., 2024)	CC0	44	44	44	0
SRPStudy	Social Reward and Nonsocial Reward Processing Across the Adult Lifespan: An Interim Multi-echo fMRI and Diffusion Dataset (Smith, Sharp, et al., 2024)	CC0	106	106	0	101

Continued on next page

Supplementary Table S1 – continued from previous page

Identifier	Dataset Name	License	Subjects	T1w	T2w	Flair
SSBStudy	The Stockholm Sleepy Brain Study: Effects of Sleep Deprivation on Cognitive and Emotional Processing in Young and Old (Tamm et al., 2020)	CC0	83	162	83	0
SSMD	SixthSense (Zadbood et al., 2022)	CC0	57	57	0	0
SUDMEX1	SUDMEX_CONN: The Mexican dataset of cocaine use disorder patients. (Garza-Villarreal et al., 2017)	CC0	144	144	0	0
SUDMEX2	SUDMEX_TMS (Angeles-Valdez et al., 2024)	CC0	53	155	0	0
TCHCC	Thalamocortical contributions to hierarchical cognitive control (Chen et al., 2024)	CC0	59	59	0	0
TCS	Truecrime (Rominger et al., 2024)	CC0	133	133	0	0
TDCP	Transdiagnostic Connectome Project (Chopra et al., 2024)	CC0	238	238	238	0
TDRS	Triple Dissociation Revisited (Van et al., 2022)	CC0	58	58	0	0
TLAMNS	Training of loss aversion modulates neural sensitivity toward potential gains	CC0	58	58	0	0
TLED	Temporal Lobe Epilepsy - UNAM (Fajardo-Valdez et al., 2024)	CC0	66	66	0	0
TUWM	Tidying Up White Matter: Neuroplastic Transformations in Sensorimotor Tracts following Slackline Skill Acquisition (Koschutnig et al., 2024)	CC0	53	159	0	0
UCLAC	UCLA Consortium for Neuropsychiatric Phenomics LA5c Study (Gorgolewski et al., 2017)	CC0	262	262	0	0
URPL	Unexplained Repeated Pregnancy Loss is Associated with Altered Perceptual and Brain Responses to Men's Body-Odor (Rozenkrantz et al., 2020)	CC0	55	55	0	0
V4CStudy	V4 crowding	CC0	49	55	42	0
VASP	Visual and audiovisual speech perception associated with increased functional connectivity between sensory and motor regions (Peelle et al., 2022)	CC0	60	60	60	0
VTIS	Valenced tactile information is evoked by neutral visual cues following emotional learning (Ehlers et al., 2024)	CC0	21	21	0	0
WMHCI	Working memory in healthy and schizophrenic individuals (Repovš and Barch, 2012)	CC0	99	99	0	0

Continued on next page

Supplementary Table S1 – continued from previous page

Identifier	Dataset Name	License	Subjects	T1w	T2w	Flair
WMRC	Working Memory and Reward in Children with and without Attention Deficit Hyperactivity Disorder (ADHD) (Lytle et al., 2020)	CC0	76	76	0	0
WRSP	A fMRI neuroimaging dataset of word reading with semantic and phonological localizers in children and adolescents	CC0	83	85	0	0
WUStudy	Washington University 120 (Power et al., 2013)	CC0	120	120	0	0

1.2 Scanner Hardware and Magnetic Field Strengths

Supplementary Table S2 summarizes the MRI scanner hardware used across the 160 datasets in the BrainScape. The table provides statistics for each imaging modality (T1w, T2w, FLAIR), organized by MRI scanner manufacturer, scanner model, and magnetic field strength (in tesla). These scanner statistics highlight the hardware heterogeneity across the datasets and enable downstream studies interested in generalization across different scanning hardware.

Supplementary Table S2: Distribution of scanner manufacturers and field strength across modalities

Modality	Manufacturer	Model	Field (T)	Count	Missing	Total
Flair		Discovery Mr750	3.0	176	2452	6552
		Signa Excite	3.0	32		
		Signa Hdx	3.0	109		
	GE	Discovery Mr750	3.0	241		
	GE	Signa Hdxt	1.5	18		
	PHILIPS			804		
	PHILIPS	Achieva	1.5	219		
	PHILIPS	Achieva	3.0	119		
	PHILIPS	Ingenia Ambition X	1.5	860		
	PHILIPS	Ingenia Elition X	3.0	190		
	PHILIPS	Intera	1.5	73		
	SIEMENS	Prisma	3.0	101		
	SIEMENS	Prisma_Fit	3.0	633		
	SIEMENS	Symphony	1.5	14		
	SIEMENS	Trio	3.0	41		
	SIEMENS	Triotim	3.0	164		

Continued on next page

Continued from previous page

Modality	Manufacturer	Model	Field (T)	Count	Missing	Total
	SIEMENS	Verio	3.0	306		
T1W			3.0	399	14451	31411
		Discovery Mr750	3.0	188		
		Signa Excite	3.0	38		
		Signa Hdx	3.0	139		
	GE	Discovery Mr750	3.0	558		
	GE	Discovery_Mr750	3.0	539		
	GE	Signa Excite	3.0	85		
	GE	Signa Hdxt	1.5	18		
	GE	Signa Premier	3.0	21		
	GE	Signa_Excite	1.5	73		
	GE	Signa_Hdxt	3.0	13		
	GE	Signa_Pet_Mr	3.0	50		
	GE	Signa_Premier	3.0	58		
	GE MEDICAL SYSTEMS	Discovery Mr750	3.0	231		
	GENERAL ELECTRICS	Discovery Mr750	3.0	102		
	PHILIPS			800		
	PHILIPS		3.0	175		
	PHILIPS	Achieva	1.5	219		
	PHILIPS	Achieva	3.0	325		
	PHILIPS	Achieva	7.0	57		
	PHILIPS	Achieva Dstream	3.0	50		
	PHILIPS	Ingenia	3.0	451		
	PHILIPS	Ingenia Ambition X	1.5	860		
	PHILIPS	Ingenia Cx	3.0	4		
	PHILIPS	Ingenia Elition X	3.0	259		
	PHILIPS	Ingenia_Cx	3.0	54		
	PHILIPS	Intera	1.5	73		
	PHILIPS	Intera	3.0	71		
	PHILIPS	Mr 7700	3.0	40		
	SIEMENS			506		
	SIEMENS		3.0	129		
	SIEMENS		3.0	60		
	SIEMENS	Aera	1.5	23		
	SIEMENS	Avanto	1.5	86		
	SIEMENS	Biograph_Mmr	3.0	28		
	SIEMENS	Magnetom Prisma	3.0	89		
	SIEMENS	Magnetom Prisma Fit	3.0	54		
	SIEMENS	Magnetom Trio	3.0	120		
	SIEMENS	Magnetom Vida	3.0	133		
	SIEMENS	Magnetom_Vida	3.0	51		
	SIEMENS	Medspec_4T	4.0	1290		
	SIEMENS	Prisma	3.0	1994		
	SIEMENS	Prisma_Fit	3.0	2429		
	SIEMENS	Skyra	3.0	1136		
	SIEMENS	Symphony	1.5	48		
	SIEMENS	Trio	3.0	204		
	SIEMENS	Trio	4.0	11		
	SIEMENS	Trio Magnetom	3.0	79		

Continued on next page

Continued from previous page

Modality	Manufacturer	Model	Field (T)	Count	Missing	Total
	SIEMENS	Triotim	3.0	2372		
	SIEMENS	Verio	3.0	168		
T2W	GE	Discovery Mr750	3.0	247	3702	7150
	GE	Discovery_Mr750	3.0	130		
	GE MEDICAL SYSTEMS	Discovery Mr750	3.0	83		
	PHILIPS	Ingenia	3.0	66		
	SIEMENS			309		
	SIEMENS	Prisma	3.0	631		
	SIEMENS	Prisma_Fit	3.0	959		
	SIEMENS	Skyra	3.0	205		
	SIEMENS	Triotim	3.0	592		
	SIEMENS	Verio	3.0	226		

References

Absher, J., Goncher, S., Newman-Norlund, R., Perkins, N., Yourganov, G., Vargas, J., Sivakumar, S., Parti, N., Sternberg, S., Teghipco, A., et al. (2024). The stroke outcome optimization project: Acute ischemic strokes from a comprehensive stroke center. *Scientific Data*, *11*(1), 839.

Alexander, L. M., Escalera, J., Ai, L., Andreotti, C., Febre, K., Mangone, A., Vega-Potler, N., Langer, N., Alexander, A., Kovacs, M., et al. (2017). An open resource for transdiagnostic research in pediatric mental health and learning disorders. *Scientific data*, *4*(1), 1–26.

Aliko, S., Huang, J., Gheorghiu, F., Meliss, S., & Skipper, J. I. (2020). A naturalistic neuroimaging database for understanding the brain using ecological stimuli. *Scientific Data*, *7*(1), 347.

Angeles-Valdez, D., Rasgado-Toledo, J., Villicaña, V., Davalos-Guzman, A., Almanza, C., Fajardo-Valdez, A., Alcala-Lozano, R., & Garza-Villarreal, E. A. (2024). The mexican dataset of a repetitive transcranial magnetic stimulation clinical trial on cocaine use disorder patients: Sudmex tms. *Scientific Data*, *11*(1), 408.

Arbula, S., Pisanu, E., & Rumiati, R. I. (2021). Representation of social content in dorsomedial prefrontal cortex underlies individual differences in agreeableness trait. *NeuroImage*, *235*, 118049.

Babayan, A., Erbey, M., Kumral, D., Reinelt, J. D., Reiter, A. M., Röbbig, J., Schaare, H. L., Uhlig, M., Anwander, A., Bazin, P.-L., et al. (2019). A mind-brain-body dataset of mri, eeg, cognition, emotion, and peripheral physiology in young and old adults. *Scientific data*, *6*(1), 1–21.

Baid, U., Ghodasara, S., Mohan, S., Bilello, M., Calabrese, E., Colak, E., Farahani, K., Kalpathy-Cramer, J., Kitamura, F. C., Pati, S., et al. (2021). The rsna-asnr-miccai brats 2021 benchmark on brain tumor segmentation and radiogenomic classification. *arXiv preprint arXiv:2107.02314*.

Balducci, T., Rasgado-Toledo, J., Valencia, A., van Tol, M.-J., Aleman, A., & Garza-Villarreal, E. A. (2022). A behavioral and brain imaging dataset with focus on emotion regulation of women with fibromyalgia. *Scientific Data*, *9*(1), 581.

Banfi, C., Koschutnig, K., Moll, K., Schulte-Körne, G., Fink, A., & Landerl, K. (2021). Reading-related functional activity in children with isolated spelling deficits and dyslexia. *Language, Cognition and Neuroscience*, *36*(5), 543–561.

Baranger, D. A., Halchenko, Y. O., Satz, S., Ragozzino, R., Iyengar, S., Swartz, H. A., & Manelis, A. (2021). Aberrant levels of cortical myelin distinguish individuals with unipolar depression from healthy controls. *medRxiv*, 2021–02.

Bathelt, J., Rastle, K., & Taylor, J. (2024). Relationship between resting state functional connectivity and reading-related behavioural measures in 69 adults. *Neurobiology of Language*, 1–19.

Bezmaternykh, D. D., Melnikov, M. Y., Savelov, A. A., Kozlova, L. I., Petrovskiy, E. D., Natarova, K. A., & Shtark, M. B. (2021). Brain networks connectivity in mild to moderate depression: Resting state fmri study with implications to nonpharmacological treatment. *Neural plasticity*, *2021*(1), 8846097.

Bissett, P. G., Eisenberg, I. W., Shim, S., Rios, J. A. H., Jones, H. M., Hagen, M. P., Enkavi, A. Z., Li, J. K., Mumford, J. A., MacKinnon, D. P., et al. (2024). Cognitive tasks, anatomical mri, and functional mri data evaluating the construct of self-regulation. *Scientific Data*, *11*(1), 809.

Biswal, B. B., Mennes, M., Zuo, X.-N., Gohel, S., Kelly, C., Smith, S. M., Beckmann, C. F., Adelstein, J. S., Buckner, R. L., Colcombe, S., et al. (2010). Toward discovery science of human brain function. *Proceedings of the national academy of sciences*, *107*(10), 4734–4739.

Boettner, B., Browning, C. R., & Calder, C. A. (2019). Feasibility and validity of geographically explicit ecological momentary assessment with recall-aided space-time budgets. *Journal of Research on Adolescence*, *29*(3), 627–645.

Bom, M. S., Brak, A. M., Raemaekers, M., Ramsey, N. F., Vansteensel, M. J., & Branco, M. P. (2024). Large-scale fmri dataset for the design of motor-based brain-computer interfaces. *medRxiv*, 2024–07.

Boroshok, A. L., Park, A. T., Fotiadis, P., Velasquez, G. H., Tooley, U. A., Simon, K. R., Forde, J. C., Delgado Reyes, L. M., Tisdall, M. D., Bassett, D. S., et al. (2022). Individual differences in frontoparietal plasticity in humans. *npj Science of Learning*, *7*(1), 14.

Botvinik-Nezer, R., Iwanir, R., Holzmeister, F., Huber, J., Johannesson, M., Kirchler, M., Dreber, A., Camerer, C. F., Poldrack, R. A., & Schonberg, T. (2019). Fmri data of mixed gambles from the neuroimaging analysis replication and prediction study. *Scientific data*, *6*(1), 106.

Botvinik-Nezer, R., Petre, B., Ceko, M., Lindquist, M. A., Friedman, N. P., & Wager, T. D. (2024). Placebo treatment affects brain systems related to affective and cognitive processes, but not nociceptive pain. *Nature communications*, *15*(1), 6017.

Brevers, D., Baeken, C., Bechara, A., He, Q., Maurage, P., Petieau, M., Sescousse, G., Vögele, C., & Billieux, J. (2021). Increased brain reactivity to gambling unavailability as a marker of problem gambling. *Addiction Biology*, *26*(4), e12996.

Bush, K. A., James, G. A., Privratsky, A. A., Fialkowski, K. P., & Kilts, C. D. (2022). Action-value processing underlies the role of the dorsal anterior cingulate cortex in performance monitoring during self-regulation of affect. *Plos one*, *17*(8), e0273376.

Cardinale, E. M., Bezek, J., Siegal, O., Freitag, G. F., Subar, A., Khosravi, P., Mallidi, A., Peterson, O., Morales, I., Haller, S. P., et al. (2024). Multivariate assessment of inhibitory control in youth: Links with psychopathology and brain function. *Psychological Science*, *35*(4), 376–389.

Castrillon, G., Epp, S., Bose, A., Fraticelli, L., Hechler, A., Belenya, R., Ranft, A., Yakushev, I., Utz, L., Sundar, L., et al. (2023). An energy costly architecture of neuromodulators for human brain evolution and cognition. *Science advances*, *9*(50), eadi7632.

Chen, X., Leach, S. C., Hollis, J., Cellier, D., & Hwang, K. (2024). Thalamocortical contributions to hierarchical cognitive control. *bioRxiv*, 2024–06.

Chopra, S., Cocuzza, C. V., Lawhead, C., Ricard, J. A., Labache, L., Patrick, L. M., Kumar, P., Rubenstein, A., Moses, J., Chen, L., et al. (2024). The transdiagnostic connectome project: A richly phenotyped open dataset for advancing the study of brain-behavior relationships in psychiatry. *medRxiv*.

Cohen, M. S., Leong, Y. C., Ruby, K., Pape, R. A., & Decety, J. (2024). Intersubject correlations in reward and mentalizing brain circuits separately predict persuasiveness of two types of isis video propaganda. *Scientific reports*, *14*(1), 13455.

consortium, A.-2. (2012). The adhd-200 consortium: A model to advance the translational potential of neuroimaging in clinical neuroscience. *Frontiers in systems neuroscience*, *6*, 62.

Crotti, M., Koschutnig, K., & Wriessnegger, S. C. (2022). Handedness impacts the neural correlates of kinesthetic motor imagery and execution: A fmri study. *Journal of Neuroscience Research*, *100*(3), 798–826.

DeLuca, V., Rothman, J., Bialystok, E., & Pliatsikas, C. (2019). Redefining bilingualism as a spectrum of experiences that differentially affects brain structure and function. *Proceedings of the National Academy of Sciences*, *116*(15), 7565–7574.

Demidenko, M. I., Mumford, J. A., & Poldrack, R. A. (2024). Impact of analytic decisions on test-retest reliability of individual and group estimates in functional magnetic resonance imaging: A multiverse analysis using the monetary incentive delay task. *bioRxiv*, 2024–03.

Di Martino, A., O'connor, D., Chen, B., Alaerts, K., Anderson, J. S., Assaf, M., Balsters, J. H., Baxter, L., Beggiato, A., Bernaerts, S., et al. (2017). Enhancing studies of the connectome in autism using the autism brain imaging data exchange ii. *Scientific data*, *4*(1), 1–15.

Di Martino, A., Yan, C.-G., Li, Q., Denio, E., Castellanos, F. X., Alaerts, K., Anderson, J. S., Assaf, M., Bookheimer, S. Y., Dapretto, M., et al. (2014). The autism brain imaging data exchange: Towards a large-scale evaluation of the intrinsic brain architecture in autism. *Molecular psychiatry*, *19*(6), 659–667.

Di Noto, T., Marie, G., Tourbier, S., Alemán-Gómez, Y., Esteban, O., Saliou, G., Cuadra, M. B., Hagmann, P., & Richiardi, J. (2023). Towards automated brain aneurysm detection in tof-mra: Open data, weak labels, and anatomical knowledge. *Neuroinformatics*, *21*(1), 21–34.

Dobrushina, O. R., Arina, G. A., Dobrynina, L. A., Suslina, A. D., Solodchik, P. O., Belopasova, A. V., Gubanova, M. V., Sergeeva, A. N., Kremneva, E. I., & Krotenkova, M. V. (2020). The ability to understand emotions is associated with interoception-related insular activation and white matter integrity during aging. *Psychophysiology*, *57*(5), e13537.

Dobrushina, O. R., Vlasova, R. M., Rumshiskaya, A. D., Litvinova, L. D., Mershina, E. A., Sinitsyn, V. E., & Pechenkova, E. V. (2020). Modulation of intrinsic brain connectivity by implicit electroencephalographic neurofeedback. *Frontiers in human neuroscience*, *14*, 527026.

Dundon, N. M., Rizor, E., Stasiak, J., Wang, J., Sabugo, K., Villaneuva, C., Barandon, P., Bostan, A. C., Lapate, R. C., & Grafton, S. T. (2024). Dissociation of novel open loop from ventral putamen to motor areas from classic closed loop in humans ii: Task-based function. *bioRxiv*, 2024–06.

Ehlers, M. R., Kryklywy, J. H., Beukers, A. O., Moore, S. R., Forys, B. J., Anderson, A. K., & Todd, R. M. (2024). Valenced tactile information is evoked by neutral visual cues following emotional learning. *Imaging Neuroscience*, *2*, 1–16.

Fajardo-Valdez, A., Camacho-Téllez, V., Rodríguez-Cruces, R., García-Gomar, M. L., Pasaye, E. H., & Concha, L. (2024). Functional correlates of cognitive performance and working memory in temporal lobe epilepsy: Insights from task-based and resting-state fmri. *Plos one*, *19*(3), e0295142.

Feher da Silva, C., Lombardi, G., Edelson, M., & Hare, T. A. (2023). Rethinking model-based and model-free influences on mental effort and striatal prediction errors. *Nature Human Behaviour*, *7*(6), 956–969.

Feng, G., Gan, Z., Yi, H. G., Ell, S. W., Roark, C. L., Wang, S., Wong, P. C., & Chandrasekaran, B. (2021). Neural dynamics underlying the acquisition of distinct auditory category structures. *Neuroimage*, *244*, 118565.

FitzGerald, T. H., Hämmerer, D., Friston, K. J., Li, S.-C., & Dolan, R. J. (2017). Sequential inference as a mode of cognition and its correlates in fronto-parietal and hippocampal brain regions. *PLoS computational biology*, *13*(5), e1005418.

Frank, L. E., & Zeithamova, D. (2023). Evaluating methods for measuring background connectivity in slow event-related functional magnetic resonance imaging designs. *Brain and Behavior*, *13*(6), e3015.

Fynes-Clinton, S., Marstaller, L., & Burianová, H. (2019). Differentiation of functional networks during long-term memory retrieval in children and adolescents. *NeuroImage*, *191*, 93–103.

Garcia, M. B., de Hollander, G., Grueschow, M., Polania, R., Woodford, M., & Ruff, C. C. (2022). Individual risk attitudes arise from noise in neurocognitive magnitude representations. *bioRxiv*, 2022–08.

Garza-Villarreal, E. A., Chakravarty, M. M., Hansen, B., Eskildsen, S. F., Devenyi, G. A., Castillo-Padilla, D., Balducci, T., Reyes-Zamorano, E., Jespersen, S. N., Perez-Palacios, P., et al. (2017). The effect of crack cocaine addiction and age on the microstructure and morphology of the human striatum and thalamus using shape analysis and fast diffusion kurtosis imaging. *Translational psychiatry*, *7*(5), e1122–e1122.

Gera, R., Or, M. B., Tavor, I., Roll, D., Cockburn, J., Barak, S., Tricomi, E., O'Doherty, J. P., & Schonberg, T. (2023). Characterizing habit learning in the human brain at the individual and group levels: A multi-modal mri study. *NeuroImage*, *272*, 120002.

Gibson, M., Newman-Norlund, R., Bonilha, L., Fridriksson, J., Hickok, G., Hillis, A. E., den Ouden, D.-B., & Rorden, C. (2024). The aphasia recovery cohort, an open-source chronic stroke repository. *Scientific Data*, *11*(1), 981.

Gifford, A. T., Jastrzębowska, M. A., Singer, J. J., & Cichy, R. M. (2024). In silico discovery of representational relationships across visual cortex. *arXiv preprint arXiv:2411.10872*.

Glick, C. C., Gajawelli, N., Sun, Y., Badami, F., Saggar, M., & Etkin, A. (2024). Concurrent single-pulse (sp) tms/fmri to reveal the causal connectome in healthy and patient populations. *bioRxiv*, 2024–09.

Goffin, C., Sokolowski, H. M., Slipenkyj, M., & Ansari, D. (2019). Does writing handedness affect neural representation of symbolic number? an fmri adaptation study. *Cortex*, *121*, 27–43.

Gold, C. E. (2018). *Exploring the resting state neural activity of monolinguals and late and early bilinguals*. Brigham Young University.

Gorgolewski, K. J., Durnez, J., & Poldrack, R. A. (2017). Preprocessed consortium for neuropsychiatric phenomics dataset. *F1000Research*, *6*.

Grössinger, D., Fischmeister, F. P. S., Witte, M., Koschutnig, K., Ninaus, M., Neuper, C., Kober, S. E., & Wood, G. (2021). The role of superstition of cognitive control during neurofeedback training. *bioRxiv*, 2021–09.

Ito, T., Kulkarni, K. R., Schultz, D. H., Mill, R. D., Chen, R. H., Solomyak, L. I., & Cole, M. W. (2017). Cognitive task information is transferred between brain regions via resting-state network topology. *Nature communications*, *8*(1), 1027.

Kable, J. W., Caulfield, M. K., Falcone, M., McConnell, M., Bernardo, L., Parthasarathi, T., Cooper, N., Ashare, R., Audrain-McGovern, J., Hornik, R., et al. (2017). No effect of commercial cognitive training on brain activity, choice behavior, or cognitive performance. *Journal of Neuroscience*, *37*(31), 7390–7402.

Keller, K. L., Pearce, A. L., Fuchs, B., Hallisky, K., Rolls, B. J., Wilson, S. J., Geier, C., & Rose, E. J. (2023). Children with lower ratings of executive functions have a greater response to the portion size effect. *Appetite*, *186*, 106569.

Keren, H., Zheng, C., Jangraw, D. C., Chang, K., Vitale, A., Rutledge, R. B., Pereira, F., Nielson, D. M., & Stringaris, A. (2021). The temporal representation of experience in subjective mood. *Elife*, *10*, e62051.

Kliemann, D., Adolphs, R., Armstrong, T., Galdi, P., Kahn, D. A., Rusch, T., Enkavi, A. Z., Liang, D., Lograsso, S., Zhu, W., et al. (2022). Caltech conte center, a multimodal data resource for exploring social cognition and decision-making. *Scientific Data*, *9*(1), 138.

Koschutnig, K., Weber, B., & Fink, A. (2024). Tidying up white matter: Neuroplastic transformations in sensorimotor tracts following slackline skill acquisition. *Human Brain Mapping*, *45*(16), e26791.

Kwok, F. Y., Wilkey, E. D., Peters, L., Khiu, E., Bull, R., Lee, K., & Ansari, D. (2023). Developmental dyscalculia is not associated with atypical brain activation: A univariate fmri study of arithmetic, magnitude processing, and visuospatial working memory. *Human Brain Mapping*, *44*(18), 6308–6325.

Lee, T.-H., Greening, S. G., Ueno, T., Clewett, D., Ponzio, A., Sakaki, M., & Mather, M. (2018). Arousal increases neural gain via the locus coeruleus–noradrenaline system in younger adults but not in older adults. *Nature human behaviour*, *2*(5), 356–366.

Li, J., Bhattasali, S., Zhang, S., Franzluebbers, B., Luh, W.-M., Spreng, R. N., Brennan, J. R., Yang, Y., Pallier, C., & Hale, J. (2022). Le petit prince multilingual naturalistic fmri corpus. *Scientific data*, *9*(1), 530.

Li, P., & Clariana, R. B. (2019). Reading comprehension in l1 and l2: An integrative approach. *Journal of Neurolinguistics*, *50*, 94–105.

Liu, P., Lin, T., Feifel, D., & Ebner, N. C. (2022). Intranasal oxytocin modulates the salience network in aging. *NeuroImage*, *253*, 119045.

Lloyd, W. K., Morriss, J., Macdonald, B., Joanknecht, K., Nihouarn, J., & Van Reekum, C. M. (2021). Longitudinal change in executive function is associated with impaired top-down frontolimbic regulation during reappraisal in older adults. *NeuroImage*, *225*, 117488.

López-Caballero, F., Curtis, M., Coffman, B. A., & Salisbury, D. F. (2024). Is source-resolved magnetoencephalographic mismatch negativity a viable biomarker for early psychosis? *European Journal of Neuroscience*, *59*(8), 1889–1906.

Lytle, M. N., McNorgan, C., & Booth, J. R. (2019). A longitudinal neuroimaging dataset on multisensory lexical processing in school-aged children. *Scientific Data*, *6*(1), 329.

Lytle, M. N., Prado, J., & Booth, J. R. (2020). A neuroimaging dataset of deductive reasoning in school-aged children. *Data in Brief*, *33*, 106405.

Markiewicz, C. J., Gorgolewski, K. J., Feingold, F., Blair, R., Halchenko, Y. O., Miller, E., Hardcastle, N., Wexler, J., Esteban, O., Goncavles, M., et al. (2021). The openneuro resource for sharing of neuroscience data. *Elife*, *10*, e71774.

Mather, M., Huang, R., Clewett, D., Nielsen, S. E., Velasco, R., Tu, K., Han, S., & Kennedy, B. L. (2020). Isometric exercise facilitates attention to salient events in women via the noradrenergic system. *NeuroImage*, *210*, 116560.

McDonald, K. R., Broderick, W. F., Huettel, S. A., & Pearson, J. M. (2019). Bayesian nonparametric models characterize instantaneous strategies in a competitive dynamic game. *Nature communications*, *10*(1), 1808.

McDonough, I. M., Bischof, G. N., Kennedy, K. M., Rodrigue, K. M., Farrell, M. E., & Park, D. C. (2016). Discrepancies between fluid and crystallized ability in healthy adults: A behavioral marker of preclinical alzheimer's disease. *Neurobiology of aging*, *46*, 68–75.

McKay, C. C., Scheinberg, B., Xu, E. P., Kircanski, K., Pine, D. S., Brotman, M. A., Leibenluft, E., & Linke, J. O. (2024). Modeling shared and specific variances of irritability, inattention, and hyperactivity yields novel insights into white matter perturbations. *Journal of the American Academy of Child & Adolescent Psychiatry*.

Meyer, C., Padmala, S., & Pessoa, L. (2019). Dynamic threat processing. *Journal of cognitive neuroscience*, *31*(4), 522–542.

Min, J., Nashiro, K., Yoo, H. J., Cho, C., Nasseri, P., Bachman, S. L., Porat, S., Thayer, J. F., Chang, C., Lee, T.-H., et al. (2022). Emotion downregulation targets interoceptive brain regions while emotion upregulation targets other affective brain regions. *Journal of Neuroscience*, *42*(14), 2973–2985.

Miranda-Angulo, A. L., Sánchez-López, J. D., Vargas-Tejada, D. A., Hawkins-Caicedo, V., Calderón, J. C., Gallo-Villegas, J., Alzate-Restrepo, J. F., Suarez-Revelo, J. X., & Castrillón, G. (2024).

Sympathovagal quotient and resting-state functional connectivity of control networks are related to gut ruminococcaceae abundance in healthy men. *Psychoneuroendocrinology*, *164*, 107003.

Momenian, M., Ma, Z., Wu, S., Wang, C., Brennan, J., Hale, J., Meyer, L., & Li, J. (2024). Le petit prince hong kong (lpphk): Naturalistic fmri and eeg data from older cantonese speakers. *Scientific data*, *11*(1), 992.

Mueckstein, M., Görgen, K., Heinzel, S., Granacher, U., Rapp, M. A., & Stelzel, C. (2024). Multitasking practice eliminates modality-based interference by separating task representations in sensory brain regions. *Journal of Neuroscience*.

Nárai, Á., Hermann, P., Auer, T., Kemenczky, P., Szalma, J., Homolya, I., Somogyi, E., Vakli, P., Weiss, B., & Vidnyánszky, Z. (2022). Movement-related artefacts (mr-art) dataset of matched motion-corrupted and clean structural mri brain scans. *Scientific data*, *9*(1), 630.

Noad, K. N., Watson, D. M., & Andrews, T. J. (2024). Familiarity enhances functional connectivity between visual and nonvisual regions of the brain during natural viewing. *Cerebral Cortex*, *34*(7), bhae285.

Nooner, K. B., Colcombe, S. J., Tobe, R. H., Mennes, M., Benedict, M. M., Moreno, A. L., Panek, L. J., Brown, S., Zavitz, S. T., Li, Q., et al. (2012). The nki-rockland sample: A model for accelerating the pace of discovery science in psychiatry. *Frontiers in neuroscience*, *6*, 152.

Novén, M., Olsson, H., Helms, G., Horne, M., Nilsson, M., & Roll, M. (2021). Cortical and white matter correlates of language-learning aptitudes. *Human brain mapping*, *42*(15), 5037–5050.

Ntoumanis, I., Sheronova, J., Davydova, A., Dolgaleva, M., Jääskeläinen, I. P., Kosonogov, V., Shestakova, A. N., & Klucharev, V. (2024). Deciphering the neural responses to a naturalistic persuasive message. *Proceedings of the National Academy of Sciences*, *121*(43), e2401317121.

Nugent, A. C., Thomas, A. G., Mahoney, M., Gibbons, A., Smith, J. T., Charles, A. J., Shaw, J. S., Stout, J. D., Namyst, A. M., Basavaraj, A., et al. (2022). The nimh intramural healthy volunteer dataset: A comprehensive meg, mri, and behavioral resource. *Scientific Data*, *9*(1), 518.

Nussenbaum, K., & Hartley, C. A. (2021). Developmental change in prefrontal cortex recruitment supports the emergence of value-guided memory. *Elife*, *10*, e69796.

Ozono, H., Komiya, A., Kuratomi, K., Hatano, A., Fastrich, G., Raw, J. A. L., Haffey, A., Meliss, S., Lau, J. K. L., & Murayama, K. (2021). Magic curiosity arousing tricks (magiccats): A novel stimulus collection to induce epistemic emotions. *Behavior Research Methods*, *53*, 188–215.

Peelle, J. E., Spehar, B., Jones, M. S., McConkey, S., Myerson, J., Hale, S., Sommers, M. S., & Tye-Murray, N. (2022). Increased connectivity among sensory and motor regions during visual and audiovisual speech perception. *Journal of neuroscience*, *42*(3), 435–442.

Penalver, J. M., González-García, C., Palenciano, A. F., Lopez-García, D., & Ruz, M. (2024). Context-dependent neural preparation for information relevance vs. probability. *Imaging Neuroscience*, *2*, 1–21.

Perszyk, E. E., Davis, X. S., Djordjevic, J., Jones-Gotman, M., Trinh, J., Hutelin, Z., Veldhuizen, M. G., Koban, L., Wager, T. D., Kober, H., et al. (2023). Odour-imagery ability is linked to food craving, intake, and adiposity change in humans. *Nature Metabolism*, *5*(9), 1483–1493.

Philips, R., Baeken, C., Billieux, J., Harris, J. M., Maurage, P., Muela, I., Öz, İ. T., Pabst, A., Sescousse, G., Vögele, C., et al. (2024). Brain mechanisms discriminating enactive mental simulations of running and plogging. *Human brain mapping*, *45*(12), e26807.

Ponticorvo, S., Manara, R., Cassandro, E., Canna, A., Scarpa, A., Troisi, D., Cassandro, C., Cuoco, S., Cappiello, A., Pellecchia, M. T., et al. (2022). Cross-modal connectivity effects in age-related hearing loss. *Neurobiology of Aging*, *111*, 1–13.

Power, J. D., Plitt, M., Gotts, S. J., Kundu, P., Voon, V., Bandettini, P. A., & Martin, A. (2018). Ridding fmri data of motion-related influences: Removal of signals with distinct spatial and physical bases in multiecho data. *Proceedings of the National Academy of Sciences*, *115*(9), E2105–E2114.

Power, J. D., Schlaggar, B. L., Lessov-Schlaggar, C. N., & Petersen, S. E. (2013). Evidence for hubs in human functional brain networks. *Neuron*, *79*(4), 798–813.

Racey, C., Kampoureli, C., Bowen-Hill, O., Bauer, M., Simpson, I., Rae, C., Del Rio, M., Simner, J., & Ward, J. (2023). An open science mri database of over 100 synaesthetic brains and accompanying deep phenotypic information. *Scientific Data*, *10*(1), 766.

Repovš, G., & Barch, D. M. (2012). Working memory related brain network connectivity in individuals with schizophrenia and their siblings. *Frontiers in human neuroscience*, *6*, 137.

Reyes-Aguilar, A., Licea-Haquet, G., Arce, B. I., & Giordano, M. (2023). Contribution and functional connectivity between cerebrum and cerebellum on sub-lexical and lexical-semantic processing of verbs. *Plos one*, *18*(9), e0291558.

Rogers, C. S., Jones, M. S., McConkey, S., McLaughlin, D. J., & Peelle, J. E. (2023). Real-time feedback reduces participant motion during task-based fmri. *bioRxiv*.

Rogers, C. S., Jones, M. S., McConkey, S., Spehar, B., Van Engen, K. J., Sommers, M. S., & Peelle, J. E. (2020). Age-related differences in auditory cortex activity during spoken word recognition. *Neurobiology of Language*, *1*(4), 452–473.

Rojek-Giffin, M., Lebreton, M., Daunizeau, J., Fariña, A., Gross, J., & De Dreu, C. K. (2023). Learning rules of engagement for social exchange within and between groups. *Proceedings of the National Academy of Sciences*, *120*(19), e2218443120.

Rominger, C., Koschutnig, K., Fink, A., & Perchtold-Stefan, C. M. (2024). Mri resting-state signature of the propensity to experience meaningful coincidences: A functional coupling analysis. *Cerebral Cortex*, *34*(7), bhae269.

Rovai, A., Lolli, V., Trotta, N., Goldman, S., & De Tiège, X. (2024). Cvrmap—a complete cerebrovascular reactivity mapping post-processing bids toolbox. *Scientific Reports*, *14*(1), 7252.

Rozenkrantz, L., Weissgross, R., Weiss, T., Ravreby, I., Frumin, I., Shushan, S., Gorodisky, L., Reshef, N., Holzman, Y., Pinchover, L., et al. (2020). Unexplained repeated pregnancy loss is associated with altered perceptual and brain responses to men's body-odor. *Elife*, *9*, e55305.

Schmidt, A. H., & Kirwan, C. B. (2024). Memory retrieval effects as a function of differences in phenomenal experience. *Brain Imaging and Behavior*, 1–8.

Scholz, C., Chan, H.-Y., Poldrack, R. A., De Ridder, D. T., Smidts, A., & Van Der Laan, L. N. (2022). Can we have a second helping? a preregistered direct replication study on the neurobiological mechanisms underlying self-control. *Human brain mapping*, *43*(16), 4995–5016.

Schuch, F., Walger, L., Schmitz, M., David, B., Bauer, T., Harms, A., Fischbach, L., Schulte, F., Schidlowski, M., Reiter, J., et al. (2023). An open presurgery mri dataset of people with epilepsy and focal cortical dysplasia type ii. *Scientific Data*, *10*(1), 475.

Seminowicz, D. A., Burrowes, S. A., Kearson, A., Zhang, J., Krimmel, S. R., Samawi, L., Furman, A. J., Keaser, M. L., Gould, N. F., Magyari, T., et al. (2020). Enhanced mindfulness-based stress reduction in episodic migraine: A randomized clinical trial with magnetic resonance imaging outcomes. *Pain*, *161*(8), 1837–1846.

Setton, R., Mwilambwe-Tshilobo, L., Girn, M., Lockrow, A. W., Baracchini, G., Hughes, C., Lowe, A. J., Cassidy, B. N., Li, J., Luh, W.-M., et al. (2023). Age differences in the functional architecture of the human brain. *Cerebral Cortex*, *33*(1), 114–134.

Shao, X., Shou, Q., Felix, K., Ojogho, B., Jiang, X., Gold, B. T., Herting, M. M., Goldwaser, E. L., Kochunov, P., Hong, L. E., et al. (2024). Age-related decline in bbb function is more pronounced in males than females. *bioRxiv*.

Shao, X., Li, A., Chen, C., Loftus, E. F., & Zhu, B. (2023). Cross-stage neural pattern similarity in the hippocampus predicts false memory derived from post-event inaccurate information. *Nature Communications*, *14*(1), 2299.

Smith, D. V., Sharp, C. J., Dachs, A., Wyngaarden, J., Sazhin, D., Yang, Y., Kos, M., Tropea, T., Kohli, I., Clithero, J. A., et al. (2024). Social reward and nonsocial reward processing across the adult lifespan: An interim multi-echo fmri and diffusion dataset. *Data in Brief*, *56*, 110810.

Smith, D. V., Wyngaarden, J., Sharp, C. J., Sazhin, D., Zaff, O., Fareri, D., & Jarcho, J. (2024). An fmri dataset of social and nonsocial reward processing in young adults. *Data in Brief*, *53*, 110197.

Snoek, L., van der Miesen, M. M., Beemsterboer, T., Van Der Leij, A., Eigenhuis, A., & Steven Scholte, H. (2021). The amsterdam open mri collection, a set of multimodal mri datasets for individual difference analyses. *Scientific data*, *8*(1), 85.

Sokolowski, H. M., Hawes, Z., Peters, L., & Ansari, D. (2021). Symbols are special: An fmri adaptation study of symbolic, nonsymbolic, and non-numerical magnitude processing in the human brain. *Cerebral Cortex Communications*, *2*(3), tgab048.

Soler-Vidal, J., Fuentes-Claramonte, P., Salgado-Pineda, P., Ramiro, N., García-León, M. Á., Torres, M. L., Arévalo, A., Guerrero-Pedraza, A., Munuera, J., Sarró, S., et al. (2022). Brain correlates of speech perception in schizophrenia patients with and without auditory hallucinations. *PloS one*, *17*(12), e0276975.

Soreq, E., Violante, I. R., Daws, R. E., & Hampshire, A. (2021). Neuroimaging evidence for a network sampling theory of individual differences in human intelligence test performance. *Nature communications*, *12*(1), 2072.

Strike, L. T., Hansell, N. K., Chuang, K.-H., Miller, J. L., de Zubicaray, G. I., Thompson, P. M., McMahon, K. L., & Wright, M. J. (2023). The queensland twin adolescent brain project, a longitudinal study of adolescent brain development. *Scientific Data*, *10*(1), 195.

Suárez-Pellicioni, M., Lytle, M., Younger, J. W., & Booth, J. R. (2019). A longitudinal neuroimaging dataset on arithmetic processing in school children. *Scientific data*, *6*(1), 1–14.

Sunavsky, A., & Poppenk, J. (2020). Neuroimaging predictors of creativity in healthy adults. *Neuroimage*, *206*, 116292.

Surani, Z., Turesky, T. K., Sullivan, E., Shama, T., Haque, R., Islam, N., Kakon, S. H., Yu, X., Petri, W. A., Nelson III, C., et al. (2024). Examining the relationship between psychosocial adversity and inhibitory control: An fmri study of children growing up in extreme poverty. *bioRxiv*, 2024–02.

Tamm, S., Schwarz, J., Thuné, H., Kecklund, G., Petrovic, P., Åkerstedt, T., Fischer, H., Lekander, M., & Nilsonne, G. (2020). A combined fmri and emg study of emotional contagion following partial sleep deprivation in young and older humans. *Scientific reports*, *10*(1), 17944.

Taylor, P. N., Wang, Y., Simpson, C., Janiukstyte, V., Horsley, J., Leiberg, K., Little, B., Clifford, H., Adler, S., Vos, S. B., et al. (2024). The imaging database for epilepsy and surgery (ideas). *Epilepsia*.

Tétreault, P., Mansour, A., Vachon-Presseau, E., Schnitzer, T. J., Apkarian, A. V., & Baliki, M. N. (2016). Brain connectivity predicts placebo response across chronic pain clinical trials. *PLoS biology*, *14*(10), e1002570.

Tomova, L., Wang, K. L., Thompson, T., Matthews, G. A., Takahashi, A., Tye, K. M., & Saxe, R. (2020). Acute social isolation evokes midbrain craving responses similar to hunger. *Nature neuroscience*, *23*(12), 1597–1605.

Turesky, T. K., Shama, T., Kakon, S. H., Haque, R., Islam, N., Someshwar, A., Gagoski, B., Petri Jr, W. A., Nelson, C. A., & Gaab, N. (2021). Brain morphometry and diminished physical growth in bangladeshi children growing up in extreme poverty: A longitudinal study. *Developmental cognitive neuroscience*, *52*, 101029.

Valdebenito-Oyarzo, G., Martínez-Molina, M. P., Soto-Icaza, P., Zamorano, F., Figueroa-Vargas, A., Larraín-Valenzuela, J., Stecher, X., Salinas, C., Bastin, J., Valero-Cabré, A., et al. (2024). The parietal cortex has a causal role in ambiguity computations in humans. *PLoS Biology*, *22*(1), e3002452.

Van, J., Nielsen, S. E., & Kirwan, C. B. (2022). Evidence for a single rather than a triple dissociation in the medial temporal lobe: An fmri recognition memory replication study. *Neuroimage: Reports*, *2*(4), 100135.

Van Essen, D. C., Smith, S. M., Barch, D. M., Behrens, T. E., Yacoub, E., Ugurbil, K., Consortium, W.-M. H., et al. (2013). The wu-minn human connectome project: An overview. *Neuroimage*, *80*, 62–79.

Van Schuerbeek, P., Baeken, C., & De Mey, J. (2016). The heterogeneity in retrieved relations between the personality trait 'harm avoidance'and gray matter volumes due to variations in the vbm and roi labeling processing settings. *PloS one*, *11*(4), e0153865.

Velanova, K., Wheeler, M. E., & Luna, B. (2008). Maturational changes in anterior cingulate and frontoparietal recruitment support the development of error processing and inhibitory control. *Cerebral cortex*, *18*(11), 2505–2522.

Vidorreta, M., Wang, Z., Rodríguez, I., Pastor, M. A., Detre, J. A., & Fernández-Seara, M. A. (2013). Comparison of 2d and 3d single-shot asl perfusion fmri sequences. *Neuroimage*, *66*, 662–671.

Visser, R. M., Henson, R. N., & Holmes, E. A. (2022). A naturalistic paradigm to investigate postencoding neural activation patterns in relation to subsequent voluntary and intrusive recall of distressing events. *Biological Psychiatry: Cognitive Neuroscience and Neuroimaging*, *7*(10), 960–969.

Wahlheim, C. N., Christensen, A. P., Reagh, Z. M., & Cassidy, B. S. (2021). Connectome-based modeling of mnemonic discrimination in younger and older adults. *bioRxiv*, 2021–07.

Wylie, K. P., Kluger, B. M., Medina, L. D., Holden, S. K., Kronberg, E., Tregellas, J. R., & Buard, I. (2023). Hippocampal, basal ganglia and olfactory connectivity contribute to cognitive impairments in parkinson's disease. *European Journal of Neuroscience*, *57*(3), 511–526.

Yau, W.-Y. W., Zubieta, J.-K., Weiland, B. J., Samudra, P. G., Zucker, R. A., & Heitzeg, M. M. (2012). Nucleus accumbens response to incentive stimuli anticipation in children of alcoholics: Relationships with precursive behavioral risk and lifetime alcohol use. *Journal of Neuroscience*, *32*(7), 2544–2551.

Zadbood, A., Nastase, S., Chen, J., Norman, K. A., & Hasson, U. (2022). Neural representations of naturalistic events are updated as our understanding of the past changes. *Elife*, *11*, e79045.

Zareba, M. R., Fafrowicz, M., Marek, T., Beldzik, E., Oginska, H., & Domagalik, A. (2022). Late chronotype is linked to greater cortical thickness in the left fusiform and entorhinal gyri. *Biological Rhythm Research*, *53*(10), 1626–1638.

Zuo, X.-N., Anderson, J. S., Bellec, P., Birn, R. M., Biswal, B. B., Blautzik, J., Breitner, J., Buckner, R. L., Calhoun, V. D., Castellanos, F. X., et al. (2014). An open science resource for establishing reliability and reproducibility in functional connectomics. *Scientific data*, *1*(1), 1–13.
